# Loss of ZNF677 expression is a predictive biomarker for lymph node metastasis in Middle Eastern Colorectal Cancer

**DOI:** 10.1038/s41598-021-01869-0

**Published:** 2021-11-16

**Authors:** Abdul K. Siraj, Sandeep Kumar Parvathareddy, Nabil Siraj, Khadija Al-Obaisi, Saud M. Aldughaither, Hadeel M. AlManea, Hussah F. AlHussaini, Fouad Al-Dayel, Khawla S. Al-Kuraya

**Affiliations:** 1 Human Cancer Genomic Research, Research Center, King Faisal Specialist Hospital and Research Center, MBC#98-16, P.O. Box 3354, Riyadh, 11211 Saudi Arabia; 2 Department of Pathology, King Faisal Specialist Hospital and Research Centre, P.O. Box 3354, Riyadh, 11211 Saudi Arabia

**Keywords:** Cancer, Biomarkers

## Abstract

Zinc-finger proteins are transcription factors with a “finger-like” domain that are widely involved in many biological processes. The zinc-finger protein 677 (ZNF677) belongs to the zinc-finger protein family. Previous reports have highlighted the tumor suppressive role of ZNF677 in thyroid and lung cancer. However, its role in colorectal cancer (CRC) has not been explored. ZNF677 protein expression was analyzed by immunohistochemistry in a large cohort of 1158 CRC patients. ZNF677 loss of expression was more frequent in CRC tissues (45.3%, 525/1158), when compared to that of normal tissue (5.1%, 11/214) *(p* < 0.0001) and was associated with mucinous histology (*p* = 0.0311), advanced pathological stage (*p* < 0.0001) and lymph node (LN) metastasis (*p* = 0.0374). Further analysis showed ZNF677 loss to be significantly enriched in LN metastatic CRC compared to overall cohort (*p* = 0.0258). More importantly, multivariate logistic regression analysis showed that ZNF677 loss is an independent predictor of LN metastasis in CRC (Odds ratio = 1.41; 95% confidence interval 1.05–1.87; *p* = 0.0203).The gain- and loss-of-function studies in CRC cell lines demonstrated that loss of ZNF677 protein expression prominently increased cell proliferation, progression of epithelial-mesenchymal transition and conferred chemoresistance, whereas its overexpression reversed the effect. In conclusion, loss of ZNF677 protein expression is common in Middle Eastern CRC and contributes to the prediction of biological aggressiveness of CRC. Therefore, ZNF677 could not only serve as a marker in predicting clinical prognosis in patient with CRC but also as a potential biomarker for personalized targeted therapy.

## Introduction

Colorectal cancer (CRC) is the second most common cause of cancer related deaths worldwide^1,2^. Thus, CRC represents a huge impact on global health. CRC has become increasingly common in the Middle Eastern population^3–5^. In Saudi Arabia, CRC is the most common cancer affecting Saudi males^6^. Lack of proper screening surveillance in this population might explain the rise in incidence and the aggressive nature of CRC^7^. It is well known that metastasis is the main cause of death in a majority of CRC patients^8,9^. Thus, identifying biomarkers for risk prediction of early metastatic disease is urgently needed.

Zinc-finger (ZNF) proteins are transcription factors with “fingerlike” domain that are involved in a wide variety of biological processes such as cell proliferation, differentiation and apoptosis, thus maintaining tissue homeostasis^10,11^. It has been shown that zinc-finger proteins could also act as co-factors involved in cellular migration and invasion^12,13^. The zinc-finger family includes both tumor suppressor genes and oncogenes^13–15^. ZNFs are involved in all the principal pathways of cancer progression from carcinogenesis to metastasis formation^16^. Furthermore, several reports have illustrated the role of zinc-finger proteins in tumorigenesis and its prognostic value in multiple types of human cancers^12,13,17–19^. ZNF677 is a member of the Kruppel C_2_H_2_-type zinc-finger protein family which has been shown to be frequently down regulated by promoter methylation in non-small cell lung cancer (NSCLC) and thyroid cancer^20,21^. Previous studies demonstrated that ZNF677 inhibits proliferation, migration, invasion and EMT progression of thyroid cancer cells^21,22^. We also reported that ZNF677 exerted its effect via modulation of EMT and activation of the AKT cascade^22^. However, its role in CRC remains unclear.

In this study, the relevance of ZNF677 expression was analyzed using immunohistochemical staining in a large cohort (1158 patient tissues) of Middle Eastern CRC and explored the functional role of ZNF677 in CRC cell lines. Our in vitro results demonstrated that ZNF677 regulates cell growth, chemoresistance and epithelial-mesenchymal transition (EMT) in CRC cells. Moreover, we explored the correlation between ZNF677 expression and clinico-pathological parameters and sought to determine if ZNF677 could be utilized as a biomarker to predict lymph node metastasis in CRC.

## Results

### Patient characteristics

Median age of the study population was 56 years (range 12–96 years) with an almost equal distribution among males and females. Majority of the tumors were of non-mucinous histology (88.9%) and were moderately differentiated (78.9%). Tumors were predominantly located in the left colon (79.7%). 12.1% (140/1158) of patients presented with distant metastasis at diagnosis. 9.4% (109/1158) of tumors showed deficient mismatch repair by immunohistochemistry (Table 1).

**Table 1:** Clinicopathological variables for the patient cohort (n = 1158).

Clinico-pathological parameter	n (%)
**Age**	
Median	56.0
Range (IQR)	47.5–67.3
**Gender**	
Male	613 (52.9)
Female	545 (47.1)
**Histological subtype**	
Adenocarcinoma	1029 (88.9)
Mucinous carcinoma	129 (11.1)
**Histological grade**	
Well differentiated	109 (9.4)
Moderately differentiated	914 (78.9)
Poorly differentiated	110 (9.5)
Unknown	25 (2.2)
**Tumor site**	
Left	923 (79.7)
Right	215 (18.6)
Unknown	20 (1.7)
**pT**	
T1	50 (4.3)
T2	185 (16.0)
T3	787 (68.0)
T4	116 (10.0)
Unknown	20 (1.7)
**pN**	
N0	599 (51.7)
N1–2	540 (46.7)
Nx	19 (1.6)
**pM**	
M0	1001 (86.4)
M1	140 (12.1)
Mx	17 (1.5)
**TNM stage**	
I	185 (16.0)
II	390 (33.7)
III	443 (38.2)
IV	140 (12.1)
**MMR protein expression status**	
dMMR	109 (9.4)
pMMR	1049 (90.6)

*IQR* inter quartile range, *dMMR* deficient mismatch repair, *pMMR* proficient mismatch repair.

### ZNF677 expression and its clinico-pathological associations

**Figure 1:**
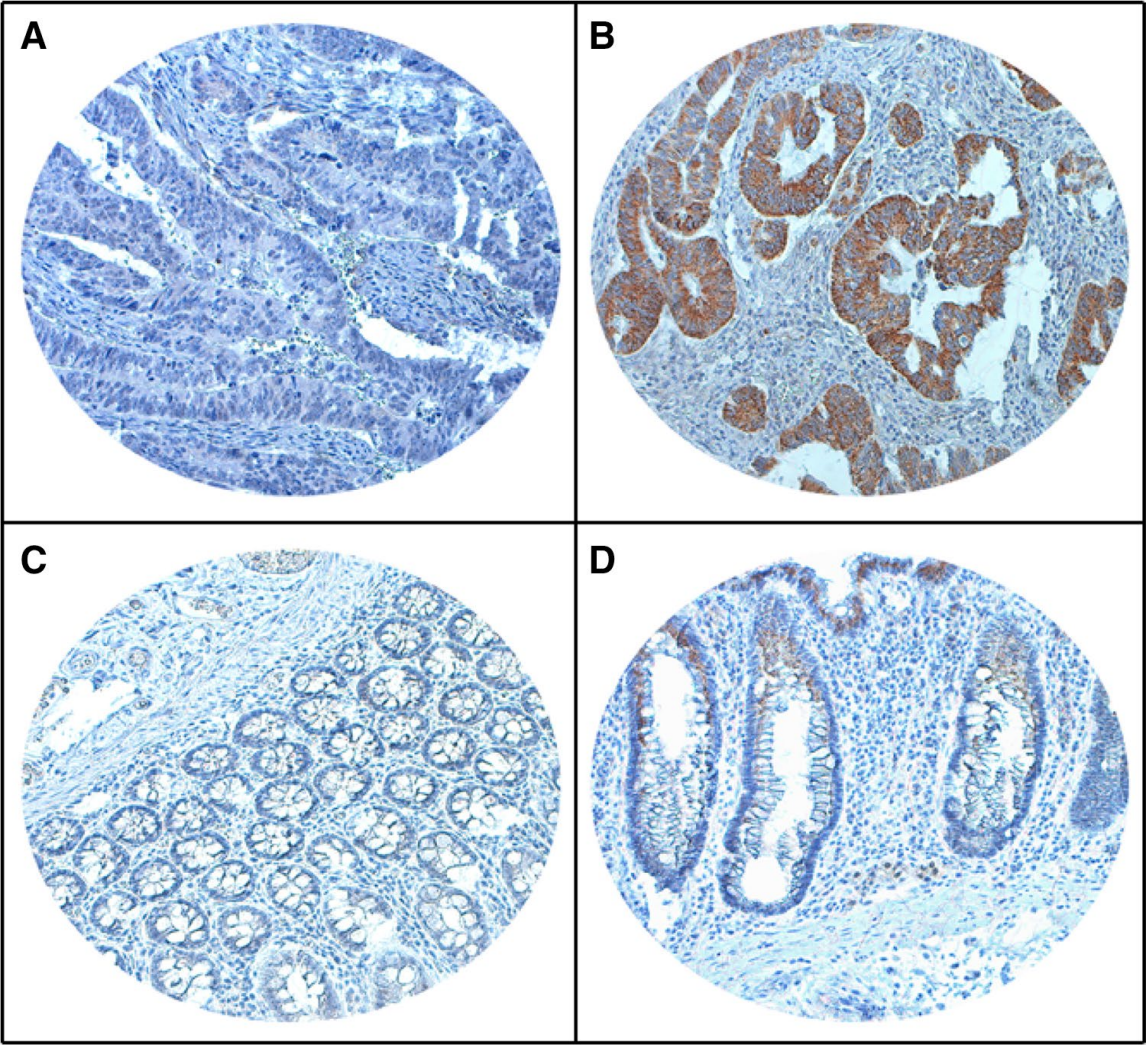
Immunohistochemical analysis of ZNF677 expression in colorectal carcinoma (CRC). Representative examples of tumors showing (**A**) loss of expression and (**B**) positive expression of ZNF677 in CRC tissues. Representative examples of normal colonic tissues showing (**C**) loss of expression and (**D**) positive expression of ZNF677. (20×/0.70 objective on an Olympus BX 51 microscope (Olympus America Inc, Center Valley, PA, USA).

**Figure 2:**
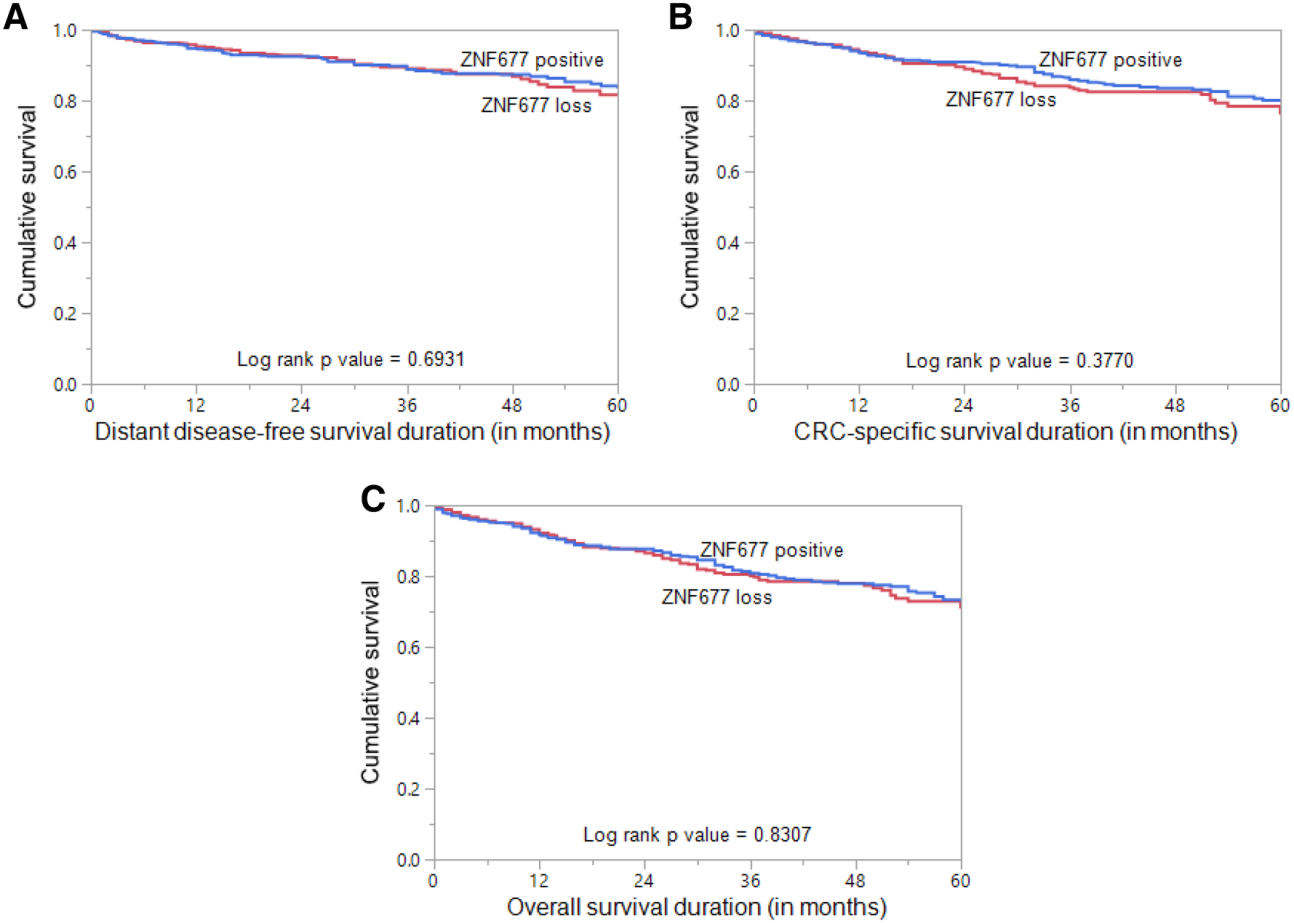
Survival analysis of ZNF677 protein expression in colorectal carcinoma (CRC). Kaplan Meier survival plot showing no statistically significant difference between ZNF677 positive and negative tumors for (**A**) distant disease-free survival (*p* = 0.6931), (**B**) CRC-specific survival (*p* = 0.3770) and (**C**) overall survival (*p* = 0.8307).

ZNF677 was primarily expressed in the cytoplasm (Fig. 1). We analysed the expression status of ZNF677 in 214 normal colonic tissues and 1158 CRC tissues using tissue microarray. ZNF677 loss was noted in 45.3% (525/1158) of CRC (Fig. 1A,B), compared to 5.1% (11/214) in normal colonic tissues (Fig. 1C,D) and this difference was statistically significant (p < 0.0001). Loss of ZNF677 expression was significantly associated with adverse clinico-pathological parameters such as mucinous histology (*p* = 0.0311), T3/4 tumors (*p* < 0.0001), lymph node metastasis (*p* = 0.0374) and advanced stage (*p* < 0.0001), whereas no association was found with carcinoembryonic antigen (CEA) levels (*p* = 0.2519), *BRAF* (*p* = 0.0748), *KRAS* (*p* = 0.3429) or *NRAS* (*p* = 0.0944) mutations (Table 2). Further analysis showed ZNF677 loss to be significantly enriched in LN metastatic CRC compared to overall cohort (51.1% vs 45.3%; *p* = 0.0258). However, ZNF677 loss was not associated with clinical outcome such as distant disease-free survival (*p* = 0.6931), CRC-specific survival (*p* = 0.3770) and overall survival (*p* = 0.8307) (Table 2; Fig. 2).

**Table 2:** Correlation of ZNF677 immunohistochemistry expression with clinico-pathological parameters in colorectal carcinoma.

	Total		ZNF677 positive		ZNF677 loss		p value
	n	%	n	%	n	%	
Total number of cases	1158		633	54.7	525	45.3	
**Age**							
≤ 50 years	380	32.8	196	51.6	184	48.4	0.1409
> 50 years	778	67.2	437	56.2	341	43.8	
**Sex**							
Male	613	52.9	346	56.4	267	43.6	0.1968
Female	545	47.1	287	52.7	258	47.3	
**Tumour site**							
Left colon	923	81.1	510	55.2	413	44.8	0.4022
Right colon	215	18.9	112	52.1	103	47.9	
**Histological type**							
Adenocarcinoma	1029	88.9	574	55.8	455	44.2	0.0311*
Mucinous carcinoma	129	11.1	59	45.7	70	54.3	
**pT**							
T1–2	235	20.7	166	70.6	69	29.4	< 0.0001*
T3–4	903	79.3	454	50.3	449	49.7	
**pN**							
N0	599	52.6	343	57.3	256	42.7	0.0374*
N1–2	540	47.4	276	51.1	264	48.9	
**pM**							
M0	1001	87.8	541	54.0	460	46.0	0.5356
M1	140	12.2	80	57.1	60	42.9	
**Tumor stage**							
I	185	16.0	131	70.8	54	29.2	< 0.0001*
II	390	33.7	198	50.8	192	49.2	
III	443	38.3	224	50.6	219	49.4	
IV	140	12.1	80	57.1	60	42.9	
**Differentiation**							
Well differentiated	109	9.6	58	53.2	51	46.8	0.4995
Moderate differentiated	914	80.7	497	54.4	417	45.6	
Poor differentiated	110	9.7	66	60.0	44	40.0	
**MMR protein expression status**							
dMMR	109	9.4	63	57.8	46	42.2	0.4888
pMMR	1049	90.6	570	54.3	479	45.7	
**CEA levels**							
< 5 ng	479	63.1	255	53.2	224	46.8	0.2519
≥ 5 ng	280	36.9	137	48.9	143	51.1	
***BRAF* mutation**							
Present	38	3.3	26	68.4	12	31.6	0.0748
Absent	1100	96.7	594	54.0	506	46.0	
***KRAS* mutation**							
Present	417	36.6	235	56.3	182	43.7	0.3429
Absent	724	63.4	387	53.4	337	46.6	
***NRAS* mutation**							
Present	33	3.2	23	69.7	10	30.3	0.0944
Absent	990	96.8	547	55.2	443	44.8	
Distant disease-free survival				83.5		81.6	0.6931
CRC-specific survival				80.1		76.1	0.3770
Overall survival				73.3		70.8	0.8307

*Significant p value.

### ZNF677 loss is an independent predictor of lymph node metastasis

Since we found ZNF677 loss to be significantly associated with lymph node metastasis, we sought to determine whether ZNF677 loss could be utilised as a predictor of lymph node metastasis. We performed multivariate logistic regression analysis and found that indeed ZNF677 loss was indeed an independent predictor for lymph node metastasis in CRC (Odds ratio = 1.41; 95% confidence interval 1.05–1.87; *p* = 0.0203) (Table 3).

**Table 3:** Multivariate analysis to assess relationship between lymph node metastasis and clinico-pathological characteristics.

Clinico-pathological variables	Odds ratio	95% CI	p-value
**Age**			
> 50 years (vs. ≤ 50 years)	0.69	0.51–0.94	0.0185*
**Gender**			
Male (vs. female)	0.97	0.73–1.29	0.8295
**Tumor side**			
Left (vs. right)	1.29	0.87–1.90	0.2063
**Histologic grade**			
Grade 3 (vs. Grade 1–2)	1.79	1.12–2.86	0.0143*
**Stage**			
IV (vs I–III)	3.27	2.16–4.96	< 0.0001*
**MMR status**			
dMMR (vs. pMMR)	0.48	0.29–0.80	0.0048*
**ZNF677 expression**			
Negative (vs. positive)	1.41	1.05–1.87	0.0203*

*Significant p value.

### ZNF677 loss of expression induces chemoresistance and promote epithelial–mesenchymal transition in colorectal cancer cells

**Figure 3:**
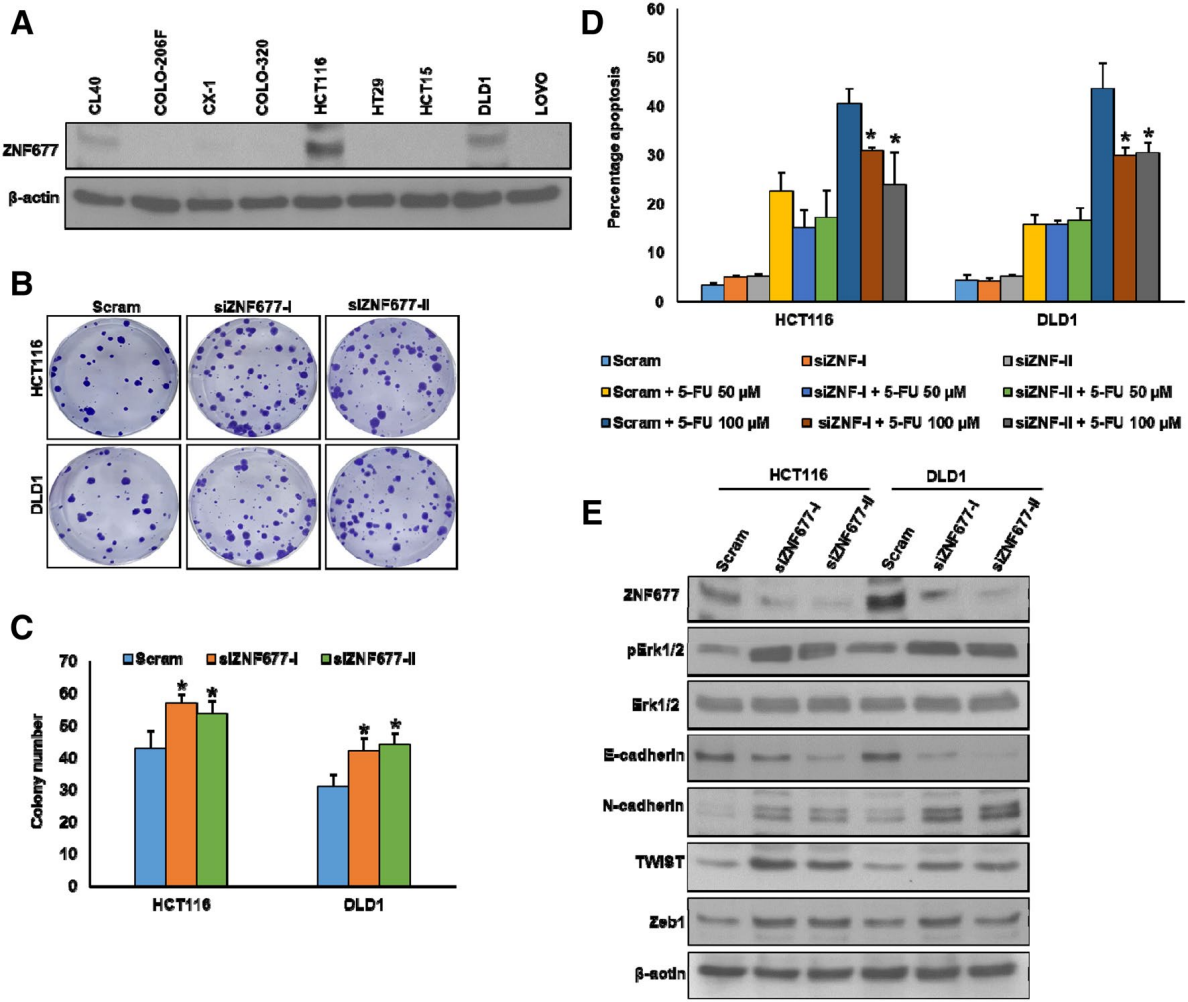
Knockdown of ZNF677 increases the chemoresistance in CRC cell lines. (**A**) Basal expression of ZNF677 in a panel of CRC cell lines. Proteins were isolated from nine CRC cell lines and immunoblotted with antibodies against ZNF677 and β-actin. (**B**, **C**) Knockdown of ZNF677 increases clonogenicity. CRC cells were transfected with scrambled siRNA and two different ZNF677 siRNA’s (20 nM). After 48 h, cells were seeded at a density of 500 cells per well in 6-well plate, and grown for an additional 10 days, then stained with crystal violet and colonies were counted (n = 3), *p < 0.05, compared to control. (**D**) Knockdown of ZNF677 increases the chemoresistance. CRC cells were transfected with either scrambled siRNA or two different ZNF677 siRNA’s (20 nM) and subsequently treated with 50 and 100 μM 5-fluorouracil (FU) for 48 h. Following treatment, cells were analysed for apoptosis by flow cytometry (n = 3), *p < 0.05, compared to respective 5-FU alone treated control. (**E**) Knockdown of ZNF677 increase the markers of cell survival and EMT progression. After transfection with ZNF677 siRNA’s, cells were immuno-blotted with antibodies against ZNF677, pERK1/2, ERK1/2, E-cadherin, N-cadherin, TWIST, Zeb1 and β-actin as indicated.

We demonstrated that the complete loss of ZNF677 protein expression in our CRC patient cohort is significantly associated with aggressive clinico-pathological parameters and lymph node metastasis. Therefore, we wanted to investigate whether the loss of ZNF677 protein expression promotes chemoresistance and epithelial-mesenchymal transition (EMT) in CRC cell lines. For the in vitro functional experimentation, we first analyzed the basal expression of ZNF677 in a panel of nine CRC cell lines by immunoblotting (Fig. 3A). On the basis of ZNF677 expression, we identified two CRC cell lines (HCT116 and DLD1) with high expression of ZNF677 and two other cell lines (COLO-320 and HT29) with low or negligible expressions for ZNF677. Next, we knocked down ZNF677 using two different siRNA sequences in ZNF677 expressing cell lines and cell growth was determined using clonogenic assay (Fig. 3B). Knockdown of ZNF677 in HCT116 and DLD1 cell lines significantly increased the cell growth (Fig. 3B,C). To study the role of ZNF677 on chemosensitivity, we knockdown ZNF677 in these cells, followed by the treatment with 5-FU for 48 h and analyzed the cells for apoptosis. As shown in Fig. 3D, both HCT116 and DLD1 cell lines became resistant to 5-FU treatment after ZNF677 knockdown as compared to CRC cells transfected with non-specific scrambled siRNA. This was further confirmed using another sequence of ZNF677 siRNA that showed similar results (Fig. 3D). We have previously reported that loss of ZNF677 expression promotes EMT and metastatic potential of papillary thyroid cancer cells^22^. Therefore, we sought to determine whether loss of ZNF677 expression has any effect on the activation of EMT in ZNF677 expressing CRC cell lines.

**Figure 4:**
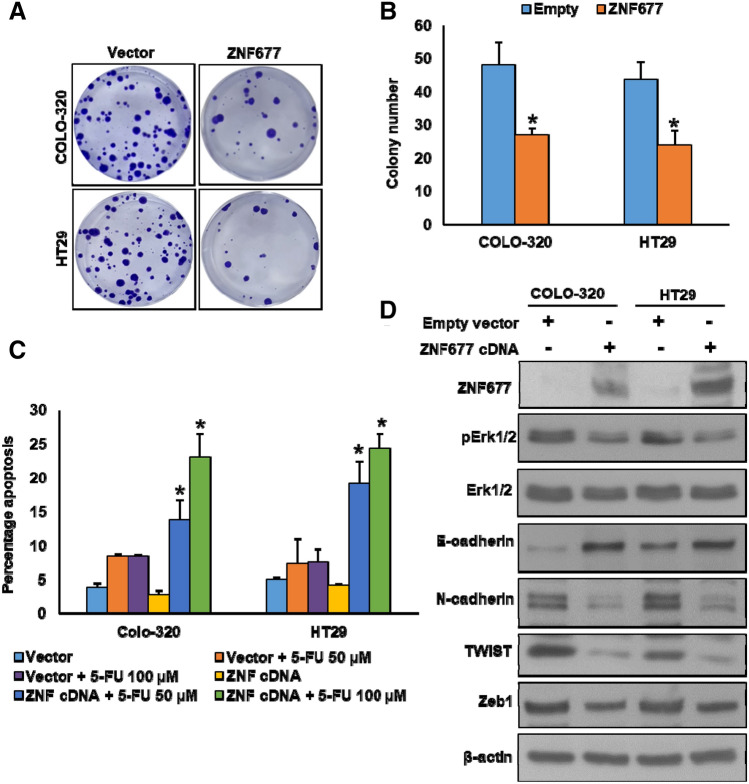
Ectopic expression of ZNF677 decreases the chemoresistance in CRC cell lines. (**A**, **B**) Overexpression of ZNF677 decreases clonogenicity. CRC cells were transfected with either empty vector or ZNF677 cDNA. After 48 h, cells were seeded at a density of 500 cells per well in 6-well plate, and grown for an additional 10 days, then stained with crystal violet and colonies were counted (n = 3), *p < 0.05, compared to control. (**C**). Overexpression of ZNF677 decreases the chemoresistance. After transfection with ZNF677 cDNA, cells were treated with 50 and 100 μM 5-fluorouracil (FU) for 48 h and analysed for apoptosis by flow cytometry (n = 3), *p < 0.05, compared to respective 5-FU alone treated control. (**D**) Overexpression of ZNF677 decrease the markers of cell survival and EMT progression. After transfection with ZNF677 cDNA, cells were immuno-blotted with antibodies against ZNF677, pERK1/2, ERK1/2, E-cadherin, N-cadherin, TWIST, Zeb1 and β-actin as indicated.

Knockdown of ZNF677 using siRNA markedly downregulated the expressions of ZNF677 and E-cadherin with an associated increase of N-cadherin, Twist, and Zeb1 expressions (Fig. 3E). We also found increase in the phosphorylation of ERK1/2, an important cell survival marker, after ZNF677 knockdown in these cell lines (Fig. 3E). Conversely, forced expression of ZNF677 in COLO-320 and HT29 cell lines decreased cell growth as shown by clonogenic assay (Fig. 4A,B). Forced expression of ZNF677 also sensitized these cells to 5-FU treatment as compared with cells transfected with empty vector (Fig. 4C). In addition, ZNF677 overexpression in COLO-320 and HT29 cell lines markedly increased ZNF677 and E-cadherin expressions, with concomitant downregulation of N-cadherin, Twist, Zeb1 and ERK1/2 phosphorylation (Fig. 4D). Taken together, these findings indicate the potential role for ZNF677 in inhibiting chemoresistance and EMT in CRC cell lines.

**Figure 5:**
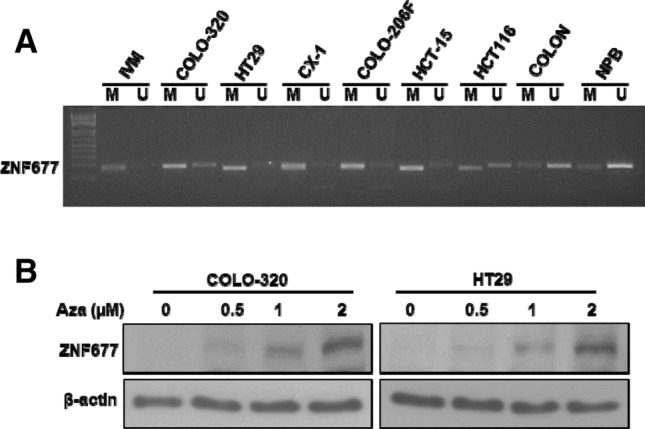
Loss of ZNF677 protein expression is associated with ZNF677 promoter methylation. (**A**) Methylation status of CRC cell lines were assessed by methylation-specific PCR for the ZNF677 gene. MSP analyses of both methylated (M) and unmethylated (U) reactions were amplified from bisulfite-treated DNA and run in a 2% agarose gel. (**B**) Demethylation of the ZNF677 gene restored ZNF677 expression in COLO-320 and HT29 cells. COLO-320 and HT29 cell lines were treated with different doses (0.5, 1, and 2 µM) of 5-aza-2′deoxycytidine for 72 h before lysis. Equal amounts of proteins were immunoblotted with antibodies against ZNF677 and β-actin.

Previous studies by us^22^ and others^20,21^ showed that ZNF677 is frequently downregulated by promoter methylation. Therefore, we sought to investigate whether loss of ZNF677 protein expression has any association with promoter methylation of the ZNF677 gene in CRC cell lines. Methylation-specific PCR (MSP-PCR) was performed to analyze the methylation status of ZNF677 promoter region in CRC cell lines. There was a complete methylation of ZNF677 gene promoter in ZNF677 low expressing cell lines, COLO-320 and HT29 cell lines (Fig. 5A). In an effort to restore methylated ZNF677, COLO-320 and HT29 cell lines were treated with 5-aza-2′-deoxycytidine, a demethylating agent for 72 h. Demethylation restored ZNF677 protein expression in COLO-320 and HT29 cell lines (Fig. 5B). These data indicate that loss of ZNF677 protein expression is associated with ZNF677 promoter methylation.

## Discussion

ZNF677, a member of KRAB zinc-finger protein family, is reported to be down-regulated by promoter methylation in NSCLC^20^ and thyroid cancer^21^. However, its role and prognostic impact in CRC has not been elucidated until now. The present study, for the first time, has demonstrated the clinical significance of ZNF677 protein expression in a large cohort of CRC patients from Middle Eastern ethnicity. Increased frequency of ZNF677 loss of expression was noted in cancer tissues when compared with control normal colorectal tissue.

Loss of ZNF677 expression was seen in 45.3% of CRCs and was significantly associated with adverse clinico-pathological factors including advanced stage and lymph node metastasis. Also, strong correlation between ZNF677 and mucinous histologic subtype was seen. Mucinous CRC is a unique histological subtype that is well known to respond poorly to chemotherapy and radiotherapy^23–26^. The association between ZNF677 protein loss and the mucinous subtype further implies the potential role of ZNF677 in clinical aggressiveness in patients with CRC.

Patients with loss of ZNF677 did not show significantly different distant disease-free survival and CRC-specific survival rates than patients exhibiting normal ZNF677 expression. However, interestingly, multivariate logistic regression analysis revealed that loss of ZNF677 expression was an independent predictor for lymph node involvement (Odds ratio = 1.41; 95% confidence interval 1.05–1.87; p = 0.0203). This suggests that loss of ZNF677 expression is a useful biomarker to identify the high risk population with potential lymph node metastasis and acts as an independent risk factor for predicting lymph node involvement in CRC patients. Given that the loss of expression of ZNF677 in CRC correlates significantly with tumor invasion (T3/4 tumors) and advanced clinical stage of the malignancy, this protein might find useful clinical application not only as a prognostic indicator but also as a potential therapeutic target for guiding personalized medicine in CRC patients. Although the immunohistochemical expression of ZNF677 has not been studied previously in CRC, studies have shown that other members of the ZNF family are associated with adverse clinico-pathological factors and poor prognosis in CRC^27–29^.

Recent studies in thyroid cancer demonstrated that ZNF677 functions as a tumor suppressor and is frequently silenced through promoter methylation^21,22^. EMT plays a critical role in regulating cancer progression, drug resistance and metastasis^30^. Our functional studies in CRC cell lines demonstrated that loss of ZNF677 expression increased cell growth, EMT progression and conferred chemoresistance, whereas forced expression of ZNF677 reversed the effect. The ERK pathway is one of the major signaling cassettes of the MAPK signaling cascade that regulate cell proliferation, cell survival and metastasis^31^. We showed that ZNF677 knockdown markedly increased ERK1/2 phosphorylation in CRC cell lines. In addition, we also confirmed that loss of ZNF677 protein expression is associated with ZNF677 promoter methylation in CRC cell line, which is in concordance with previous reports^20–22^.

Despite the strength of our study being the use of a simple technique like immunohistochemistry to evaluate a clinically and histologically well characterized large Middle Eastern CRC cohort, we acknowledge that this study has several limitations. First being the lack of methylation analysis in our tumor tissues. Although previous studies suggest that methylation is a major mechanism for transcription regulation of ZNF677 in several tumors^20,21^, additional studies are needed to demonstrate the correlation between ZNF677 down regulation and increased ZNF677 methylation in CRC to better understand the tumor suppressor function of ZNF677 in CRC. Second, this study is retrospective in nature, with heterogeneity of chemotherapy regimens among this patient cohort since samples were collected over a long duration.

To conclude, we found that loss of ZNF677 expression is frequent in Middle Eastern CRC. ZNF677 loss in CRC tissue samples correlates with positive LN status and aggressive clinico-pathological characteristics. Therefore, ZNF667 may be a novel biomarker for predicting aggressive tumor subtype in Middle Eastern CRC and may serve as a potential therapeutic target for CRC.

## Methods

### Sample selection

Archival paraffin embedded tissue samples from 1158 CRC patients diagnosed between 1990 to 2015 at King Faisal Specialist Hospital and Research Center (Riyadh, Saudi Arabia) were included in the present study. The clinico-pathological data were obtained from medical records and have been summarized in Table 1. Institutional Review Board of King Faisal Specialist Hospital and Research Centre provided ethical approval for the current study. Research Advisory Council (RAC) granted waiver of informed consent for use of retrospective patient case data under project RAC# 2170 025. All the methods were carried out in accordance with relevant guidelines and regulations.

### Tissue microarray construction and immunohistochemistry

All samples were analyzed in a tissue microarray (TMA) format. TMA construction was performed as described earlier^32^. Briefly, tissue cylinders with a diameter of 0.6 mm were punched from representative normal and tumor regions of each donor tissue block and brought into recipient paraffin block using a modified semiautomatic robotic precision instrument (Beecher Instruments, Woodland, WI, USA). Two cores of normal and CRC tissues were arrayed from each case.

Tissue microarray slides were processed and stained manually as described previous^33^. Primary antibody against ZNF677 (HPA-024796, 1:300 dilution, pH 6.0, Sigma Aldrich, St. Louis, Missouri, USA) was used. A predominantly cytoplasmic staining was observed. The proportion of positively stained cells was calculated as a percentage for each core and the scores were averaged across two tissue cores from the same tumor to yield a single percent staining score representing each cancer patient. For the purpose of statistical analysis, the scores were dichotomised. Cases showing any positive expression were classified as ZNF677 positive and those with no expression were classified as ZNF677 loss.

Mismatch repair protein staining and evaluation was done as described previously^34^. IHC scoring was done by two pathologists, blinded to the clinico-pathological characteristics. Discordant scores were reviewed together to achieve agreement.

### DNA isolation

DNA was isolated from formalin-fixed, paraffin-embedded (FFPE) tumor tissues using Gentra DNA isolation kit (Gentra, Minneapolis, MN, USA), following the manufacturer’s recommendations.

### PCR and sanger sequencing

Primer 3 software was used to design the primers for the entire coding and splicing regions of exons 1 and 2 in *KRAS* and *NRAS* and exon 15 in *BRAF* (available upon request). PCR was performed in a total volume of 25 µl with 20 ng of genomic DNA, 2.5 µl 10× Taq buffer, 2.3 mM dNTPs, 1 unit Taq polymerase and 0.2 µM each primer and de-ionized water. The efficiency and quality of the amplified PCR products was confirmed by loading them on a 2% agarose gel.

For Sanger sequencing, the PCR products were subsequently subjected to direct sequencing with BigDye terminator V 3.1 cycle sequencing reagents and analysed on an ABI 3730XL DNA analyser (Applied Biosystems, Foster City, CA). In addition, the pathogenic variants detected by Targeted capture sequencing analysis were further confirmed by Sanger sequencing analysis. Reference sequences were downloaded from NCBI GenBank. Sequencing traces were analysed with the Mutation Surveyor v4.04 (Soft Genetics, LLC, State College, PA).

### Tissue culture experiments

All human CRC cell lines; CL40, COLO-206F, CX-1, COLO-320, HCT116, HT29, HCT-15, DLD1 and LOVO were obtained from American Type Culture Collection and cultured in RPMI 1640 media supplemented with 10% fetal bovine serum (FBS), 100 U/mL penicillin, 100 U/mL streptomycin at 37 °C in a humidified atmosphere containing 5% CO_2_. All the treatments were performed in reduced FBS condition (5%). Apoptosis analysis was performed using annexin V/propidium iodide dual staining and measured by flow cytometry as previously described^22^. ZNF677 antibody was purchased from Sigma Aldrich (St. Louis, MO, USA). Antibodies against pERK1/2 (4370), ERK1/2 (4695), E-cadherin (3195), Zeb1 (3396), and β-actin (3700) were purchased from Cell Signaling Technology (Danvers, Massachusetts, USA). Antibodies against N-cadherin (ab98952) and TWIST (ab175430) were purchased from Abcam (Cambridge, Massachusetts, USA). All the original uncropped Western blot images are presented in Supplementary Fig. S1.

### Gene silencing using small interfering RNA

Cells were transfected two different sequence of ZNF677 siRNA’s (SR317571A-5′r(CCAGAAGAGAGGUAAAACUAUUGTC)3′ and SR317571B-5′r(GGAUUUACAAGCAAGGGAUUUCAC)3′ and scrambled negative control siRNA (SR30004) from OriGene (Rockville, MD, USA) using Lipofectamine 2000 (Invitrogen, Carlsbad, CA, USA) for 6 h, following which the lipid and siRNA complex was removed and fresh growth medium was added. After 48 h of transfection, cells were used for various experiments such as clonogenic assay, apoptosis analysis and immunoblotting.

### Plasmid and transfection

Plasmid DNA encoding human ZNF677 (RC207997) was purchased from OriGene (Rockville, MD, USA). Transfection was performed using Lipofectamine™2000 (Invitrogen, Carlsbad, CA) according to the manufacturer's protocol. Briefly, CRC cells were seeded in 6-well culture plates; when approximately 50% confluent, cells were transfected with 4 μg plasmid. After 48 h of transfection, cells were used for various experiments such as clonogenic assay, apoptosis analysis and immunoblotting.

### Bisulfite modification and methylation-specific PCR

Genomic DNA extracted from CRC cell lines was subjected to bisulfite modification using an EZ DNA Methylation kit (Zymo Research, Orange, CA, USA) as reported previously^35^. Methylation-specific PCR (MSP) was performed on bisulfite-treated DNA using primers specific for CpG islands in the ZNF677 promoter: forward primer, 5′-GAGGAGAGGTTCGGTAGTTC-3′ and reverse primer, 5′-TACGCGAATACACTAAAACGA-3′. For unmethylated DNA: forward primer, 5′-GTTTTTGTTGATTTGGAAGTGG-3′ and reverse primer, 5′-AACTAAAAACATCTTAAAACCACACC-3′. MSP products of ZNF677methylation and unmethylation were analyzed on 2% agarose gels and visualized under UV illumination after staining with ethidium bromide.

### Statistical analysis

Contingency table analysis and Chi square tests were used to study the relationship between clinico-pathological variables and protein expression. Kaplan–Meier method was used to generate distant disease-free, CRC-specific and overall survival curves, with Mantel–Cox log-rank test used to evaluate significance. Cox proportional hazards regression model was used to perform multivariate analysis, after adjusting for clinico-pathological variables like age, gender, stage, grade, site of tumor and MSI status. The limit of significance for all analyses was defined as p value of < 0.05; two-sided tests were used in these calculations. The JMP14.0 (SAS Institute, Inc., Cary, NC, USA) software package was used for data analyses.

For all functional studies, data presented are means ± SD of triplicates in an independent experiment, which was repeated for at least two times with the same results. Student t test (two-tailed) was performed for statistical significance, with p < 0.05 used as the cut-off.

## Supplementary Information

Supplementary Figure S1.
