# Supplementary material for: Loss of ZNF677 expression is a predictive biomarker for lymph node metastasis in Middle Eastern Colorectal Cancer
Source: Sci Rep. 2021 Nov 16;11:22346. doi: 10.1038/s41598-021-01869-0 (PMC8595636; doi:10.1038/s41598-021-01869-0)
Supplement: Supplementary file 1 — Supplementary Figure S1. [file 41598_2021_1869_MOESM1_ESM.pdf]

Supplementary Figure 1: Uncropped western blot/gel images

Figure 3A

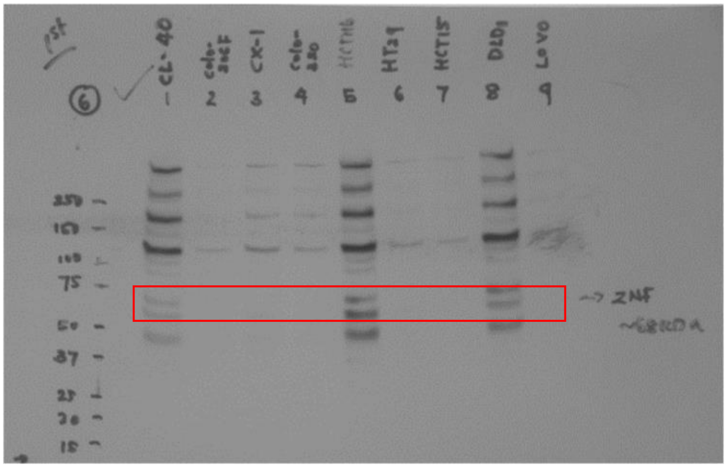

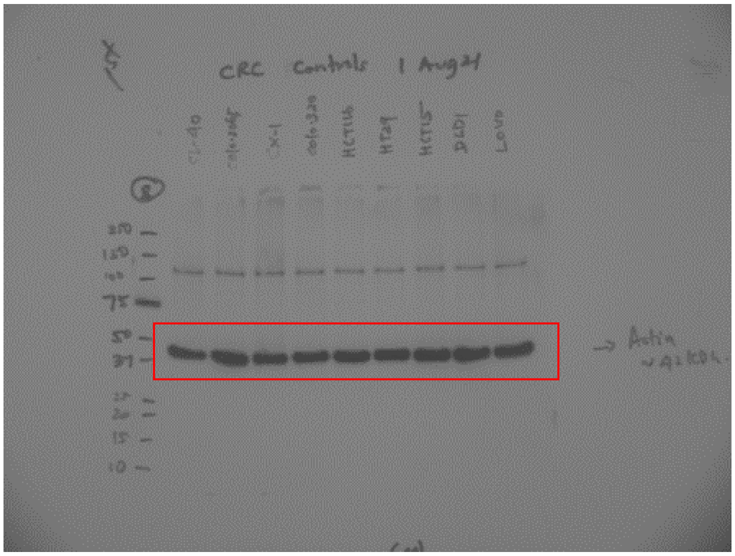

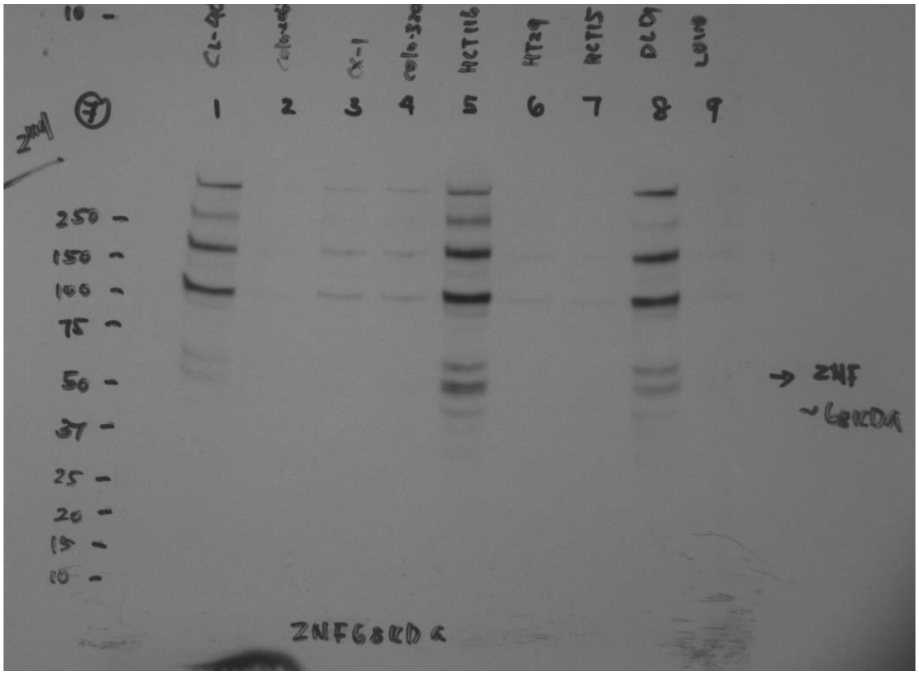

2nd
CX-1
colo-320
HCT116
HT29
HCT15
DLD1
1 2 3 4 5 6 7 8 9
250
150
100
75
50
37
25
20
15
10
→ ZNF
~68kDa
ZNF68kDa

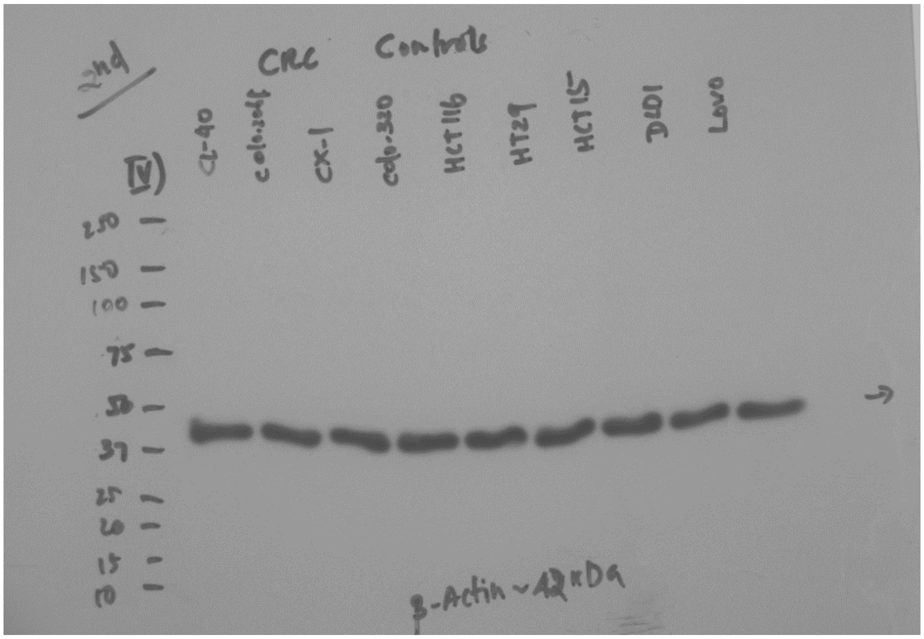

2nd
CRC Controls
IV)
CX-1
colo-320
HCT116
HT29
HCT15
DLD1
LoVo
250
150
100
75
50
37
25
20
15
10
β-Actin ~42kDa

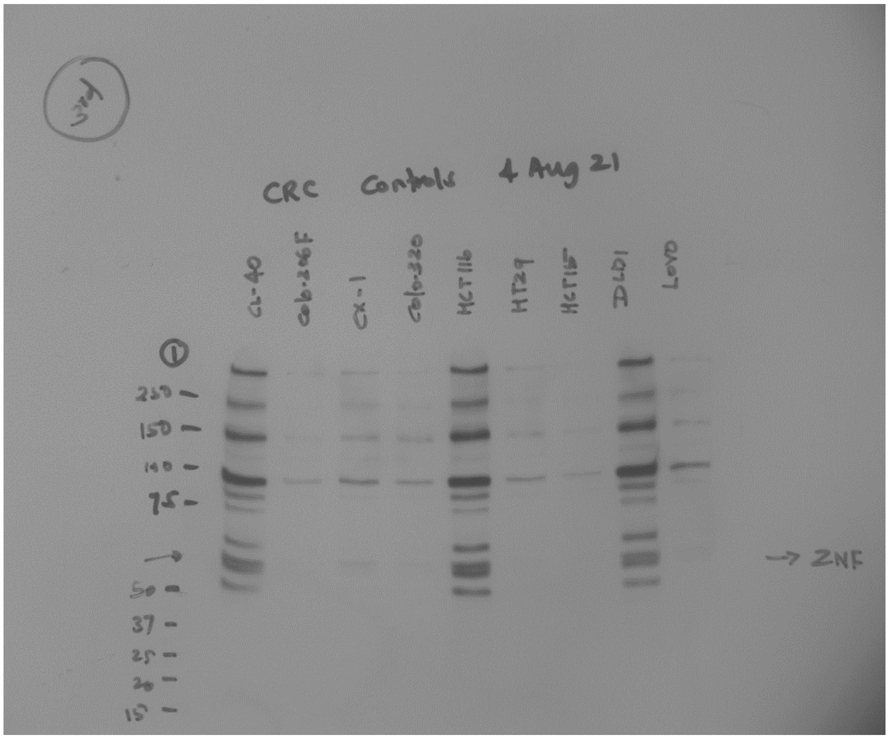
3rd
CRC Controls 4 Aug 21
CL-40
Colo-205F
CX-1
Colo-320
HCT116
HT29
HCT15
DLD1
LoVo
①
250
150
100
75
50
37
25
20
15
→ ZNF

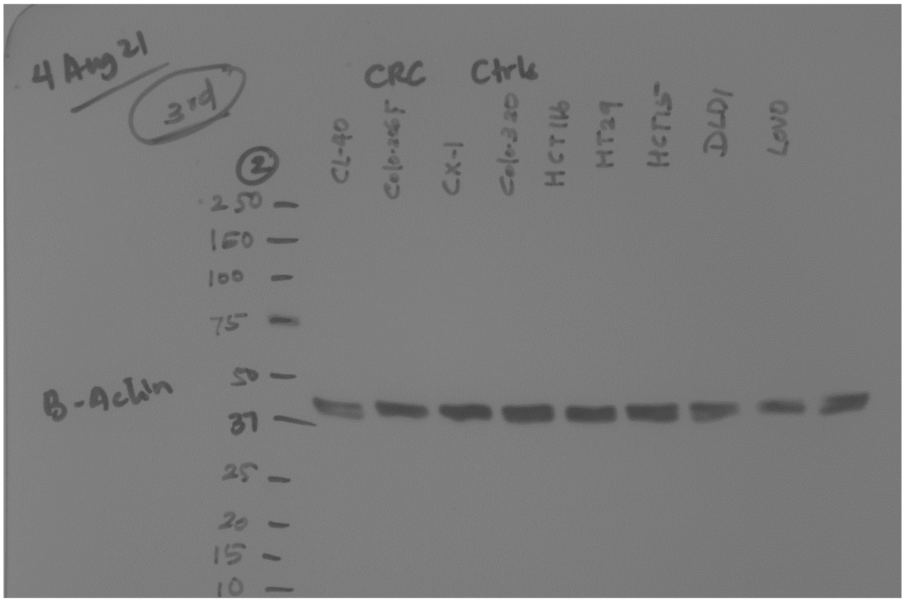
4 Aug 21
3rd
CRC Ctrls
CL-40
Colo-205F
CX-1
Colo-320
HCT116
HT29
HCT15
DLD1
LoVo
②
250
150
100
75
50
37
25
20
15
10
B-Actin

Figure 3E

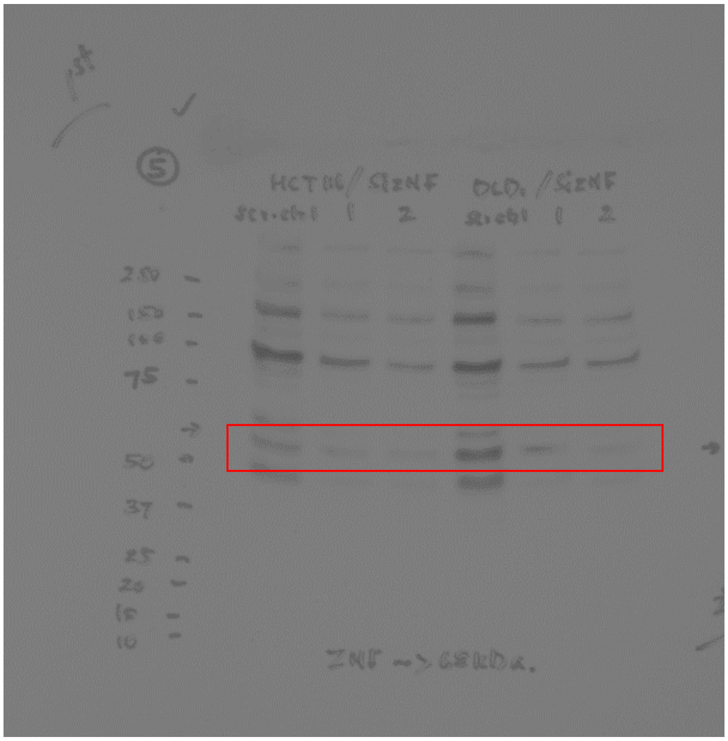
HCT116/SiZNF
DLD1/SiZNF
250
150
100
75
50
37
25
20
15
10
ZNF ~> 68kDa.

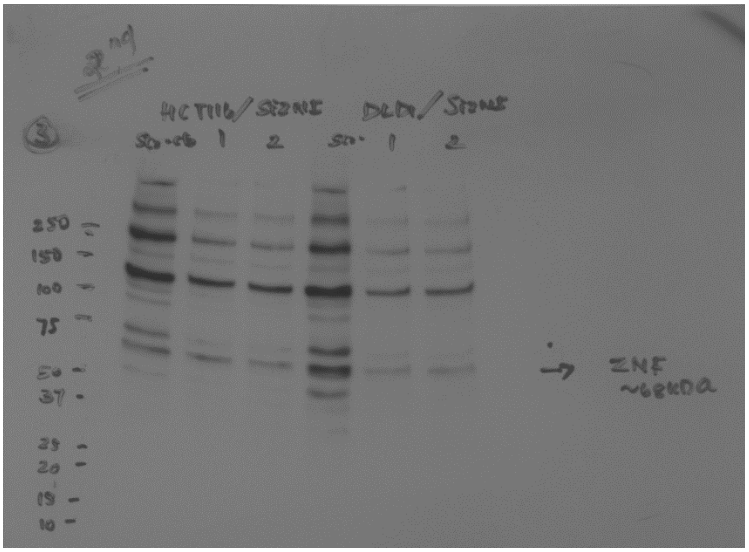
2nd
HCT116/SiZNF
DLD1/SiZNF
250
150
100
75
50
37
25
20
15
10
ZNF
~68kDa

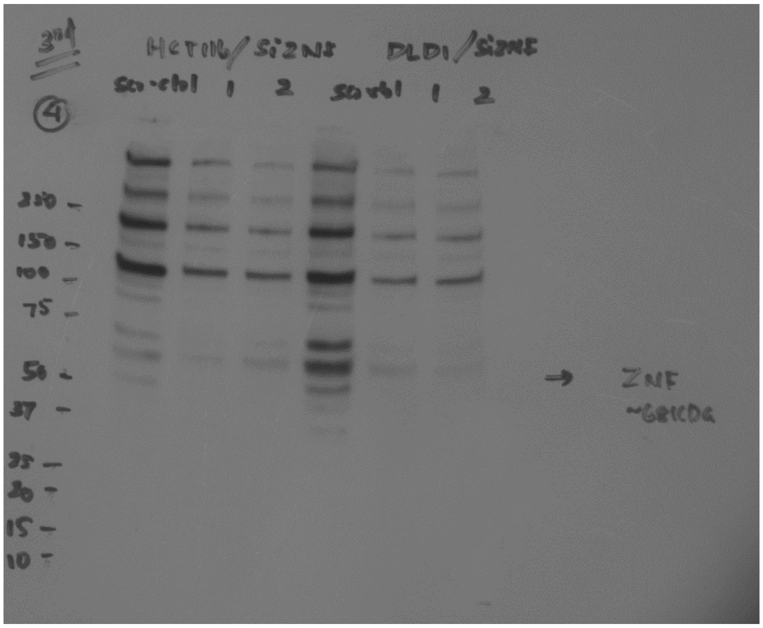

250
150
100
75
50
37
25
20
15
10
ZNF

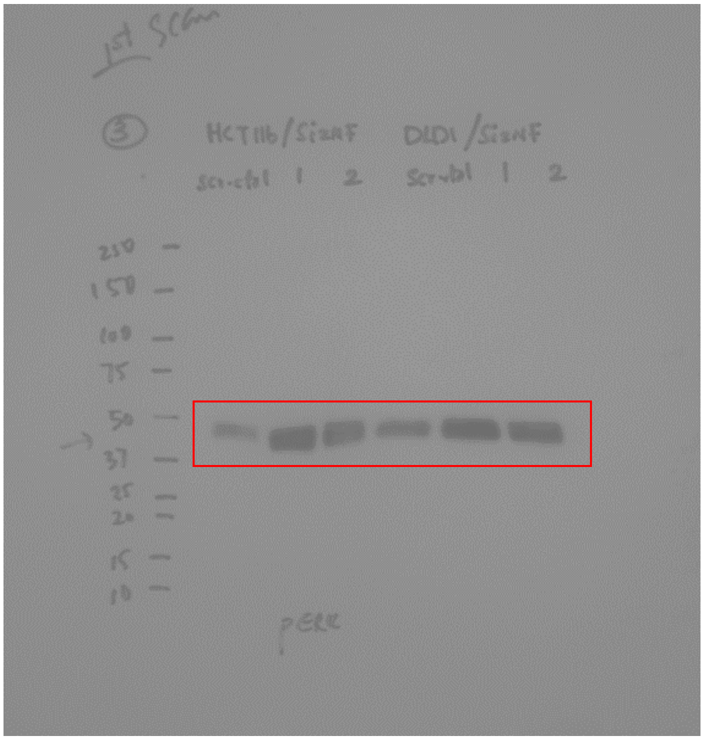

1st scan
③
250
150
100
75
50
37
25
20
15
10
pERK

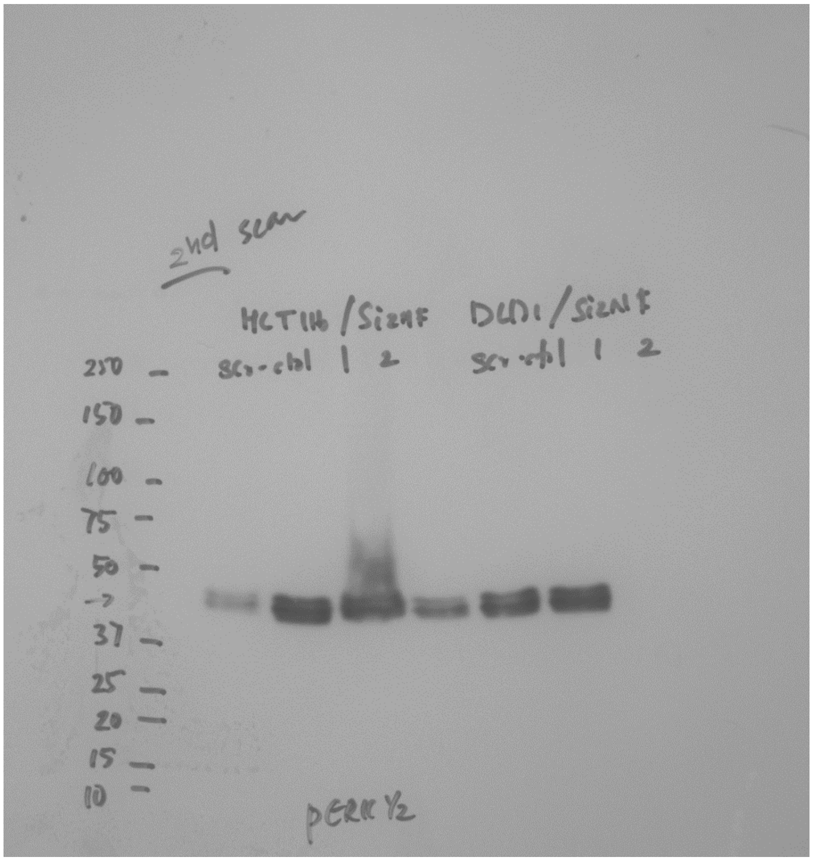
2nd scan
250
150
100
75
50
37
25
20
15
10
pERK 1/2

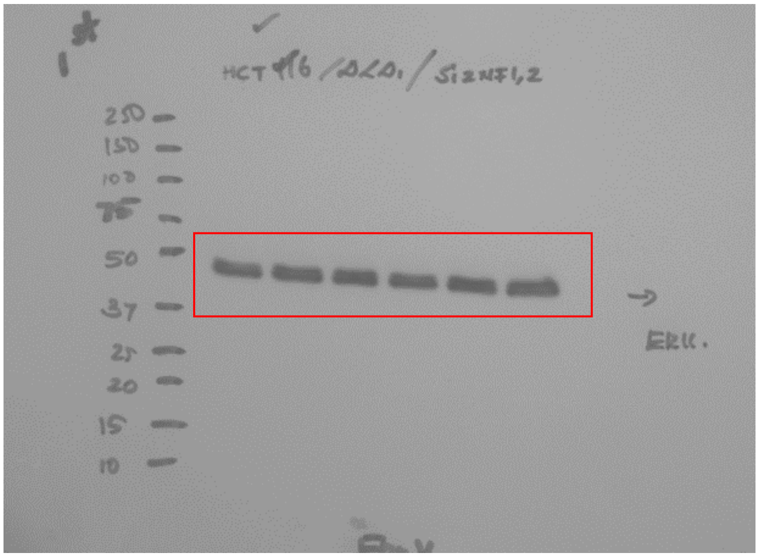
250
150
100
50
37
25
20
15
10
ERK.

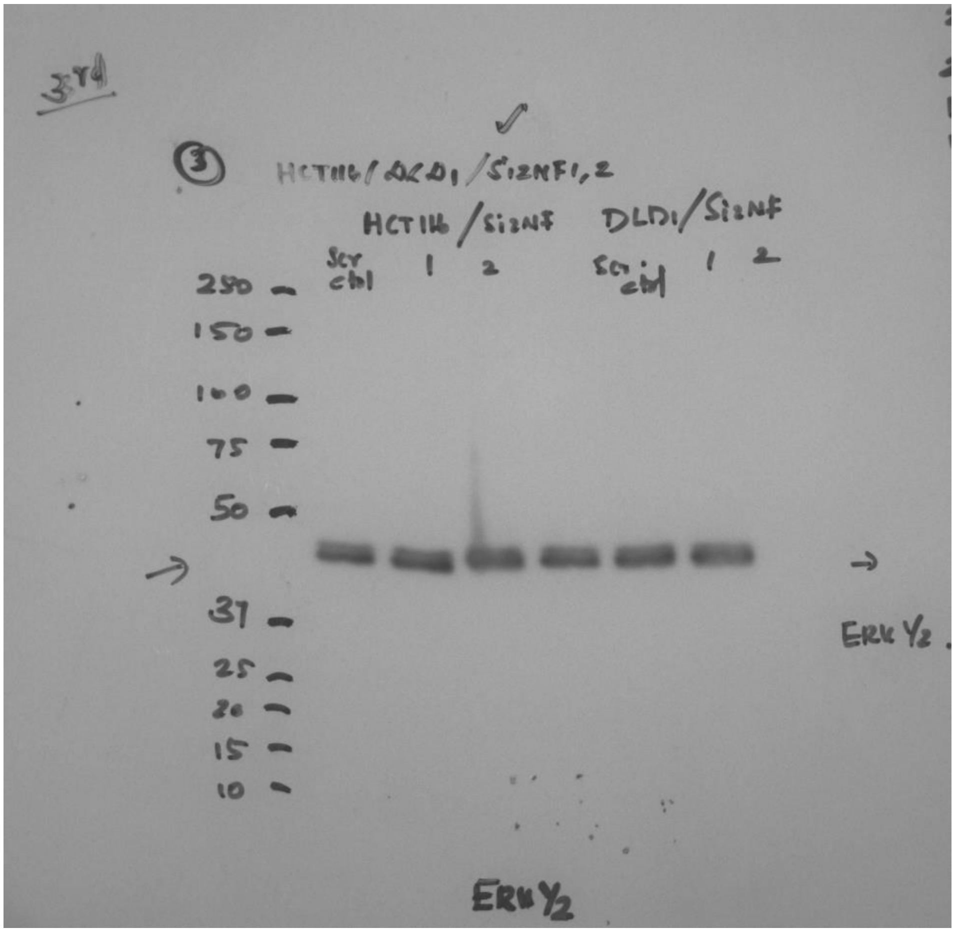
3rd
③ HCT116/DLD1/SiZNF1,2
HCT116/SiZNF DLD1/SiZNF
Scr ctrl 1 2 Scr ctrl 1 2
250
150
100
75
50
37
25
20
15
10
ERK1/2
ERK1/2

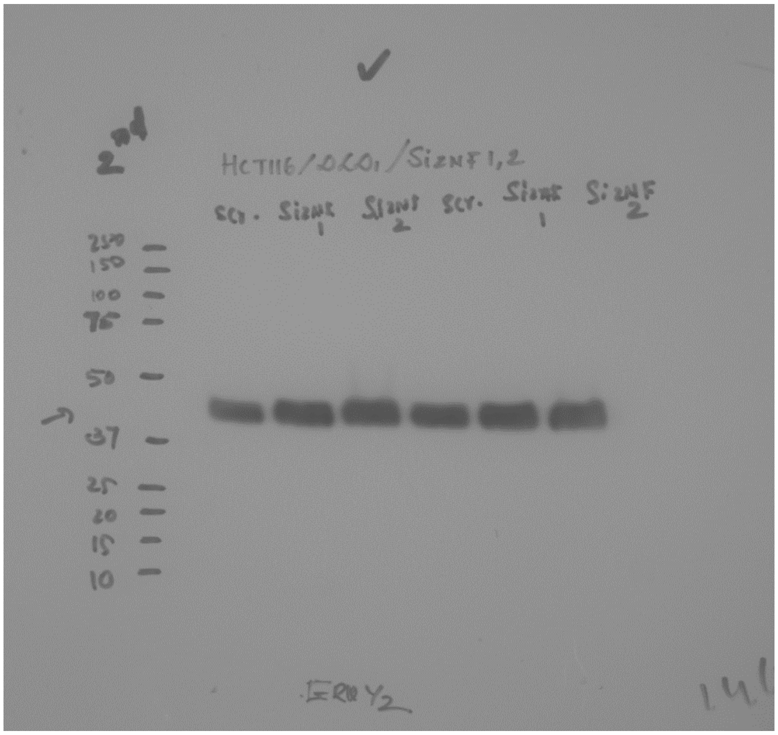
2nd
HCT116/DLD1/SiZNF1,2
Scr. SiZNF 1 SiZNF 2 Scr. SiZNF 1 SiZNF 2
250
150
100
75
50
37
25
20
15
10
ERK1/2

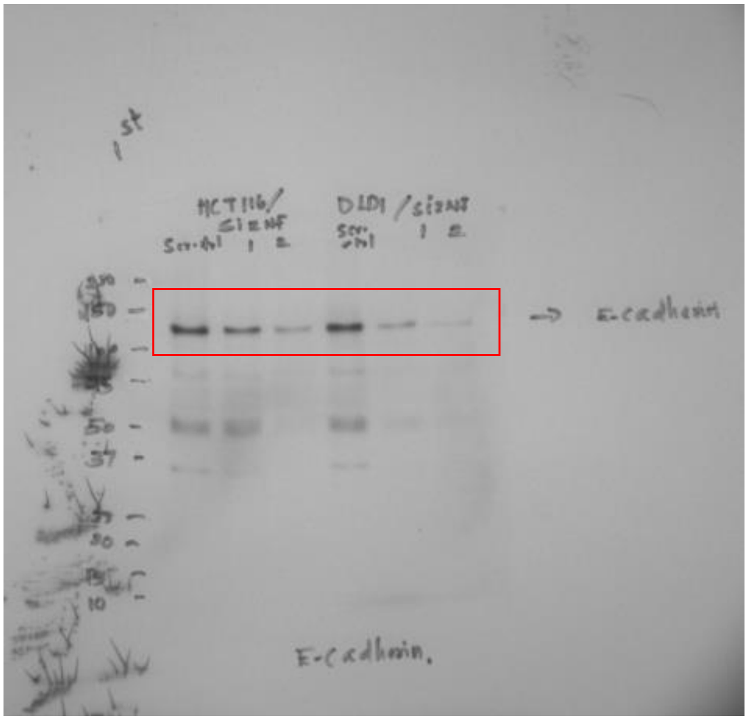
1st
HCT116/ si3NF
Scr.ctrl 1 2
DLD1 / si3NF
Scr. ctrl 1 2
→ E-cadherin
E-cadherin.

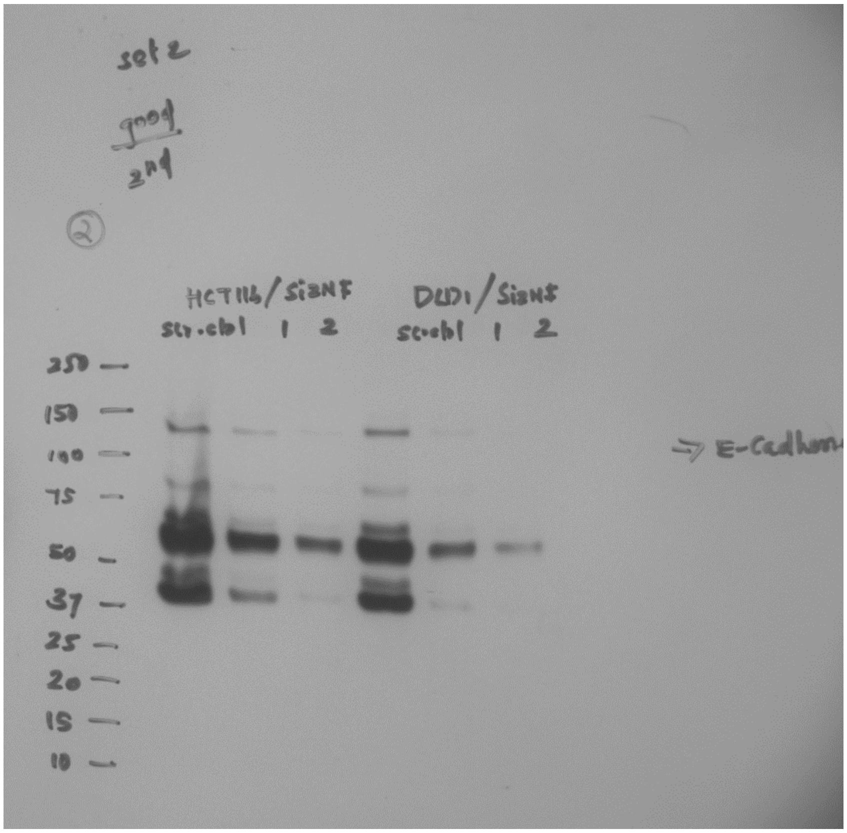
set 2
good
2nd
②
HCT116/Si3NF
Scr.ctrl 1 2
DLD1/Si3NF
Scr.ctrl 1 2
250
150
100
75
50
37
25
20
15
10
⇒ E-Cadherin

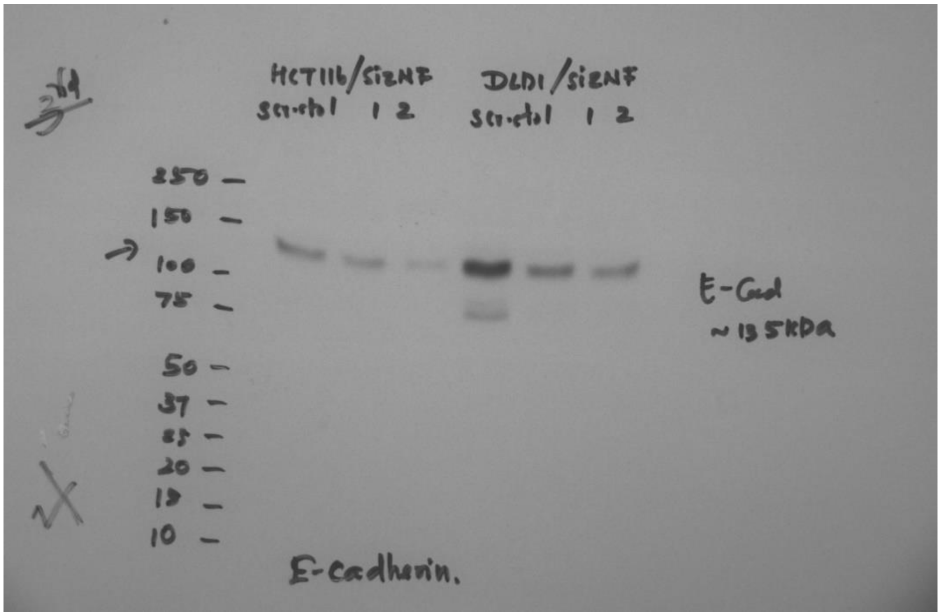
3rd
HCT116/siZNF
Scr-ctrl 1 2
DLD1/siZNF
Scr-ctrl 1 2
250
150
100
75
50
37
25
20
15
10
E-Cad
~135KDa
E-cadherin.

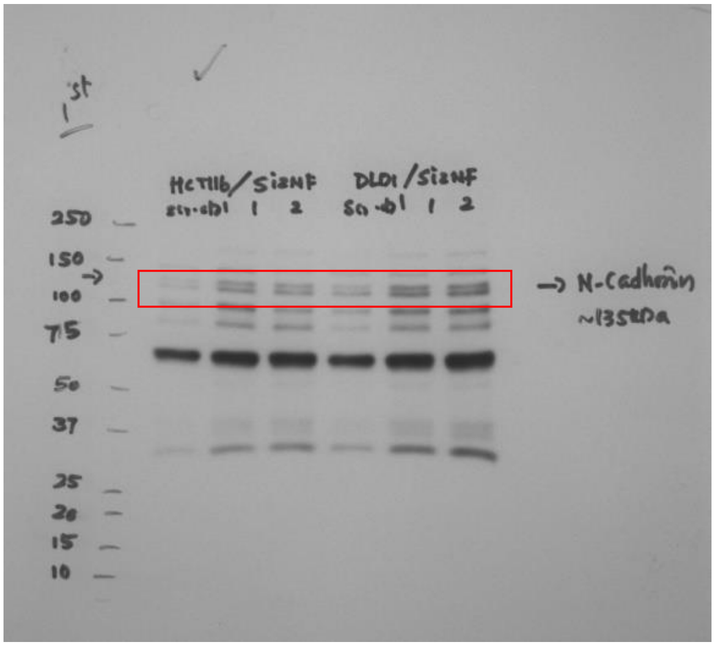
1st
HCT116/siZNF
scr-ctrl 1 2
DLD1/siZNF
Scr-ctrl 1 2
250
150
100
75
50
37
25
20
15
10
N-Cadherin
~135KDa

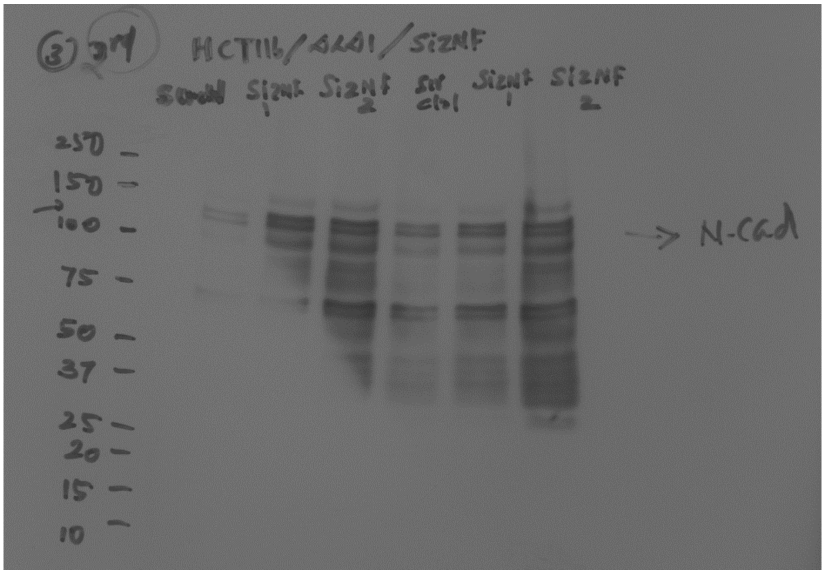
HCT116/SLC1/SiZNF
250
150
100
75
50
37
25
20
15
10
N-Cad

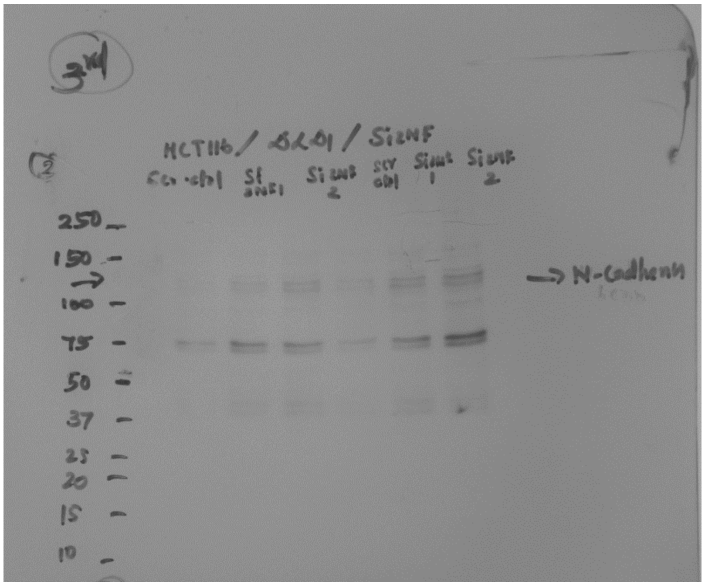
HCT116/SLC1/SiZNF
250
150
100
75
50
37
25
20
15
10
N-Cadherin

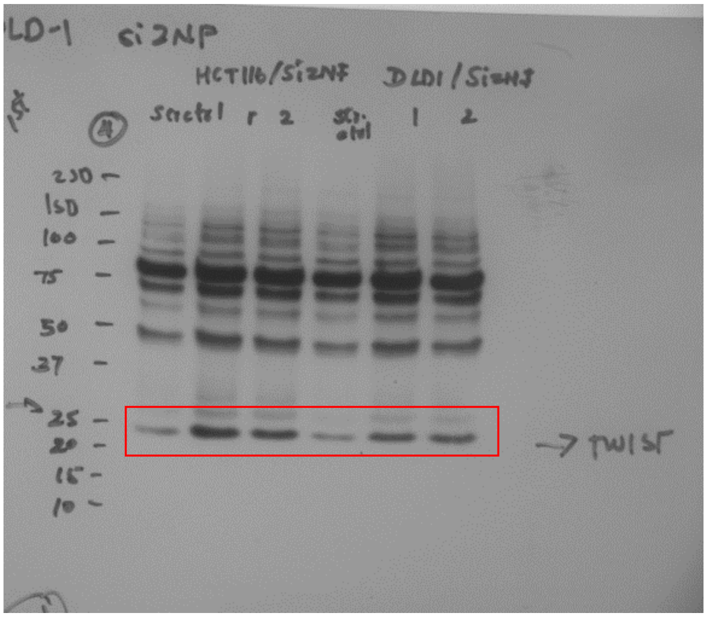
HCT116/SiZNF
250
150
100
75
50
37
25
20
15
10
→ TWIST

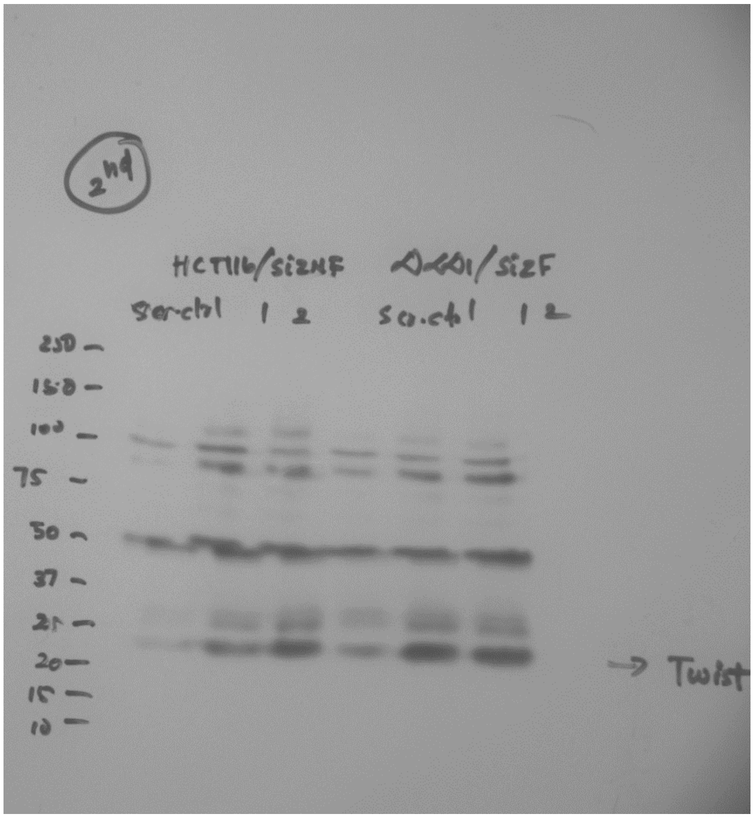
2nd
HCT116/SiZNF
250
150
100
75
50
37
25
20
15
10
→ Twist

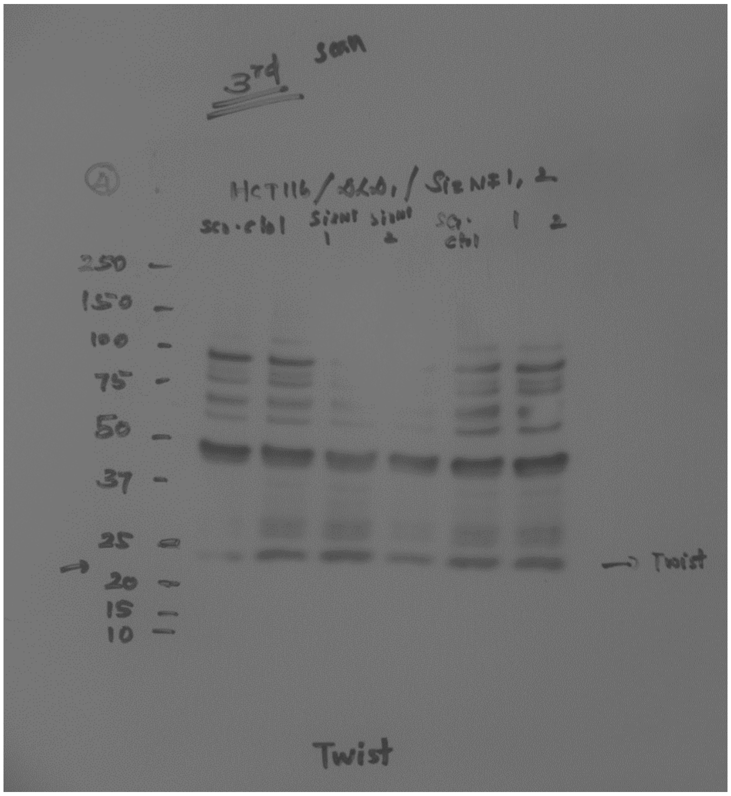
3rd scan
Twist
Twist

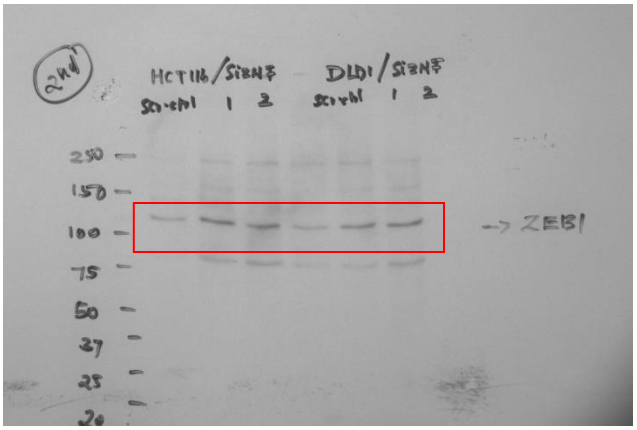
ZEB1

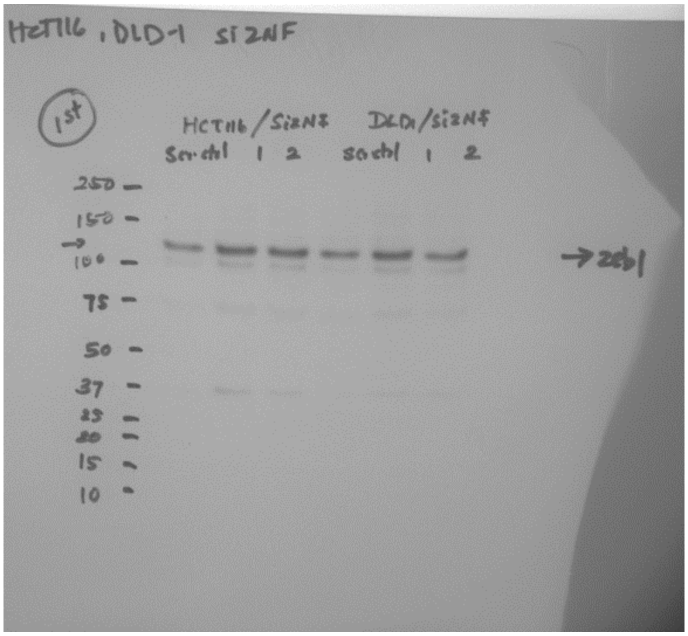
HCT116, DLD-1 si ZNF
1st
HCT116/SiZNF DLD1/SiZNF
Scr ctrl 1 2 Scr ctrl 1 2
250
150
100
75
50
37
25
20
15
10
zeb1

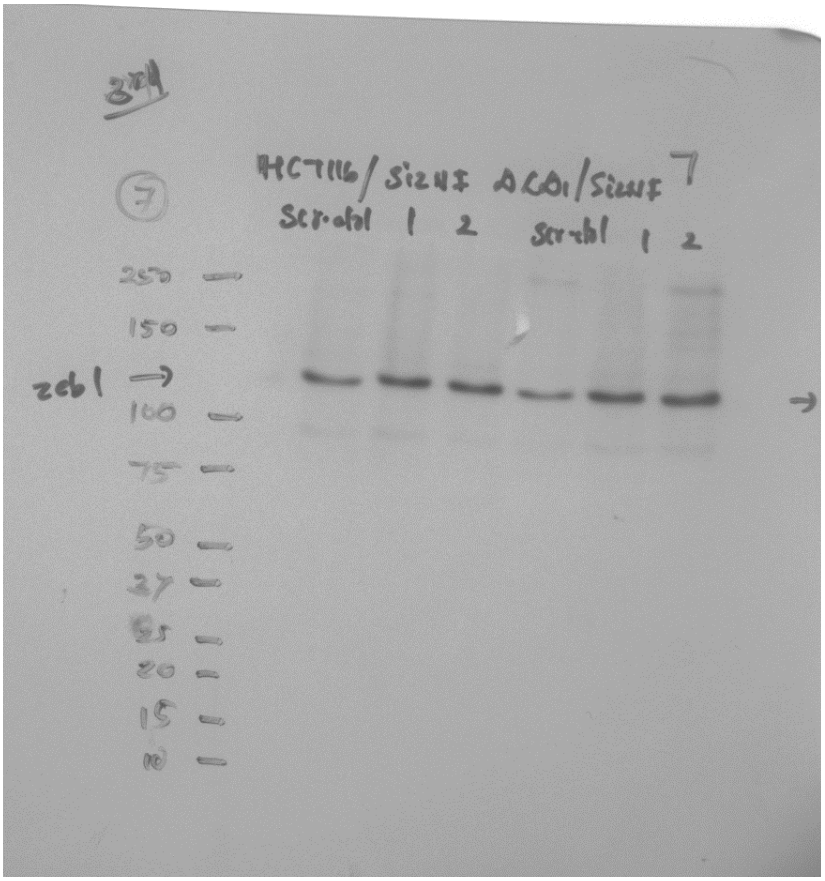
HCT116/SiZNF DLD1/SiZNF
Scr ctrl 1 2 Scr ctrl 1 2
250
150
zeb1
100
75
50
37
25
20
15
10

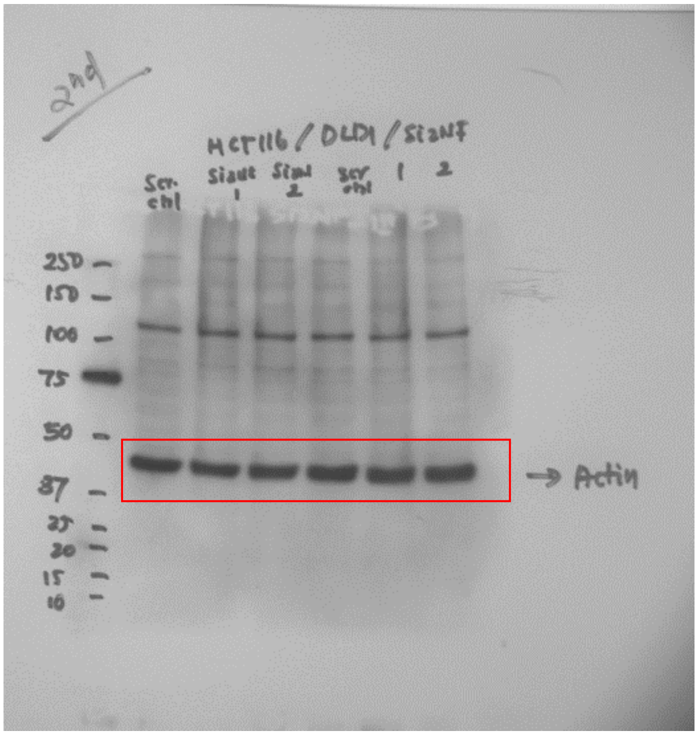
2nd
250
150
100
75
50
37
25
20
15
10
Actin

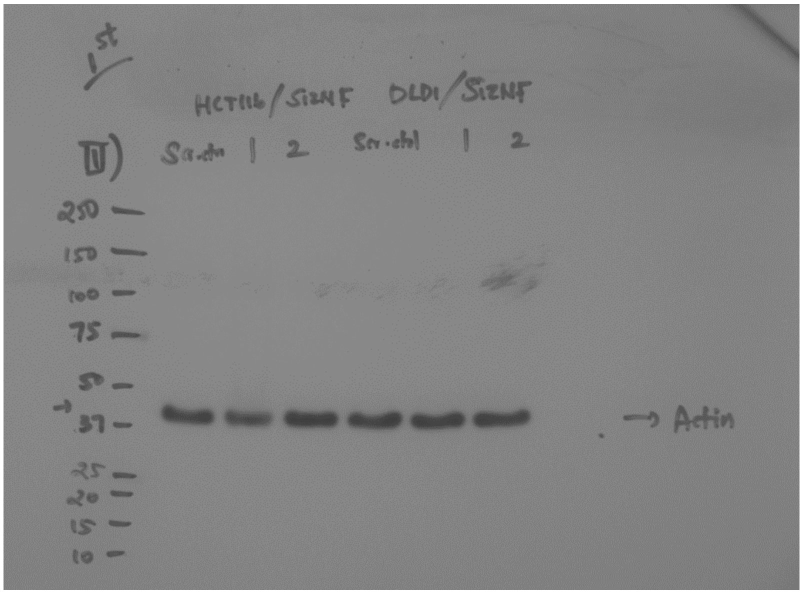
1st
1
2
250
150
100
75
50
37
25
20
15
10
Actin

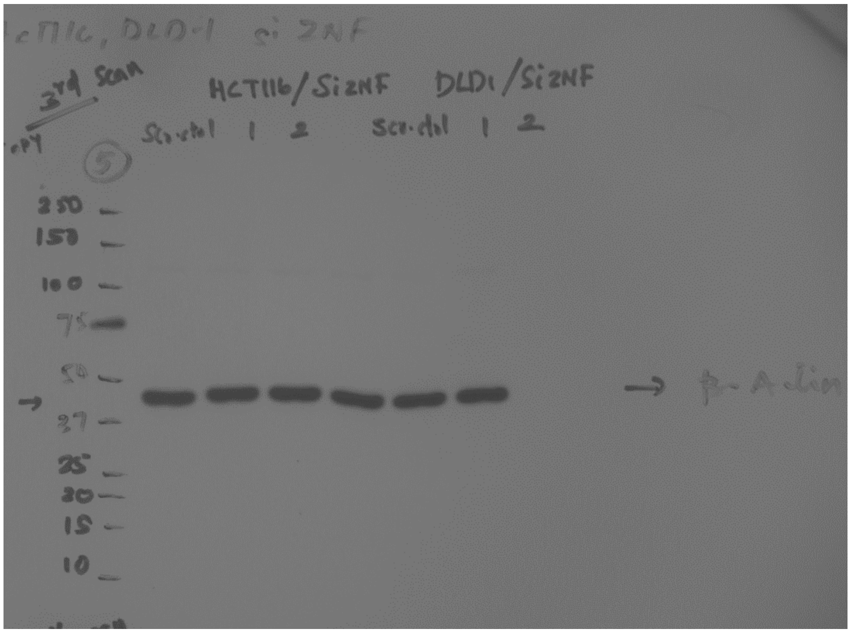
3rd scan
copy
HCT116/Si ZNF
DLD1/Si ZNF
Scr-ctrl 1 2
Scr-ctrl 1 2
250
150
100
75
50
37
25
20
15
10
β-Actin

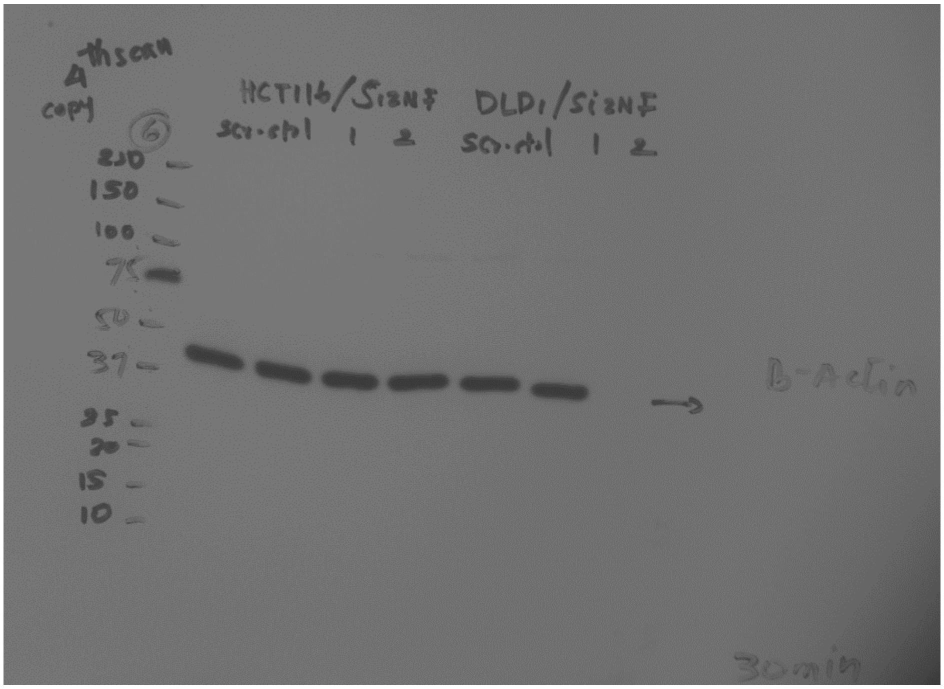
4th scan
copy
HCT116/Si ZNF
DLD1/Si ZNF
Scr-ctrl 1 2
Scr-ctrl 1 2
250
150
100
75
50
37
25
20
15
10
B-Actin
30min

Figure 4D

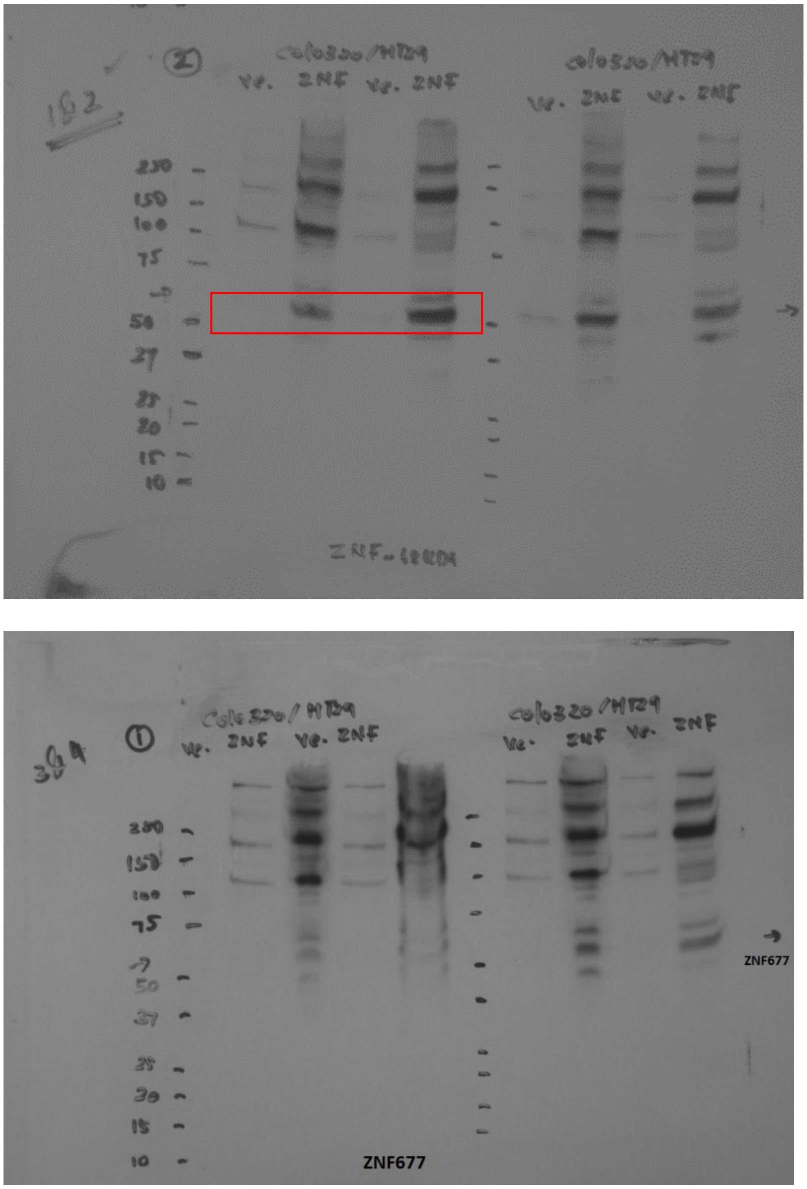

colo320/HT29
Ve. ZNF Ve. ZNF
colo320/HT29
Ve. ZNF Ve. ZNF
ZNF677
ZNF677

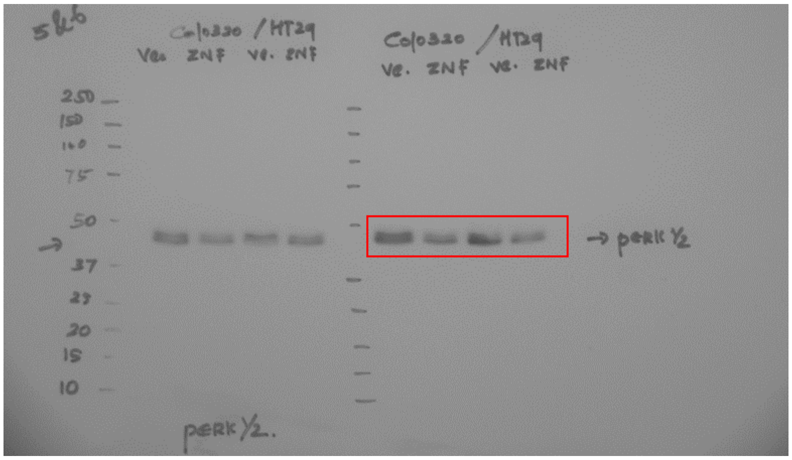
Colo320 / HT29
Ve. ZNF Ve. ZNF
Colo320 / HT29
Ve. ZNF Ve. ZNF
250
150
100
75
50
37
25
20
15
10
pERK1/2
pERK1/2.

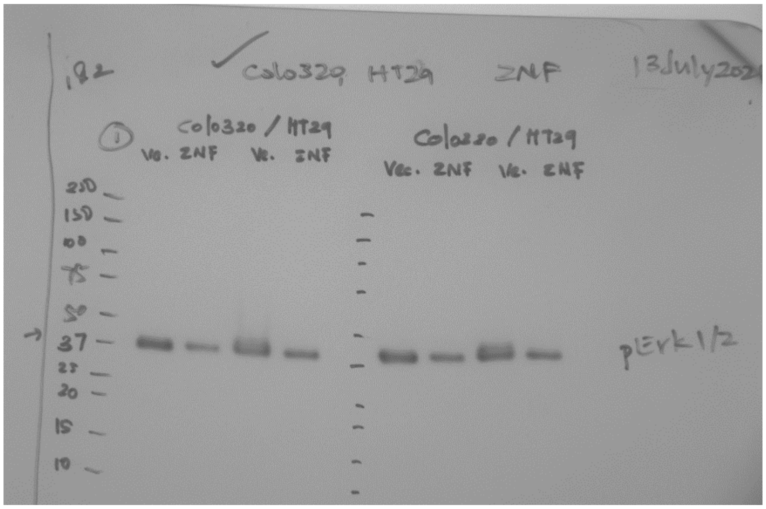
1&2
Colo320 HT29 ZNF
13July202
Colo320 / HT29
Ve. ZNF Ve. ZNF
Colo320 / HT29
Vec. ZNF Ve. ZNF
250
150
100
75
50
37
25
20
15
10
pErk1/2

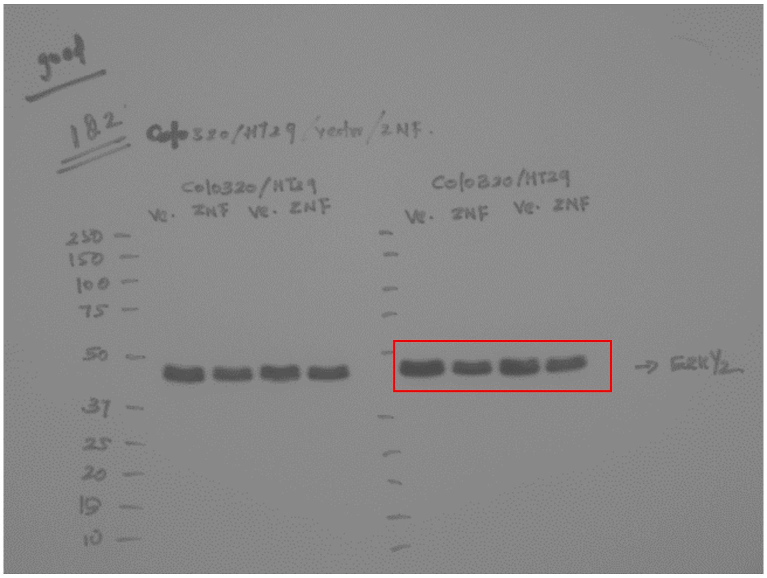
good
1&2
Colo320/HT29/vector/ZNF.
Colo320/HT29
Ve. ZNF Ve. ZNF
Colo320/HT29
Ve. ZNF Ve. ZNF
250
150
100
75
50
37
25
20
15
10
ERK1/2

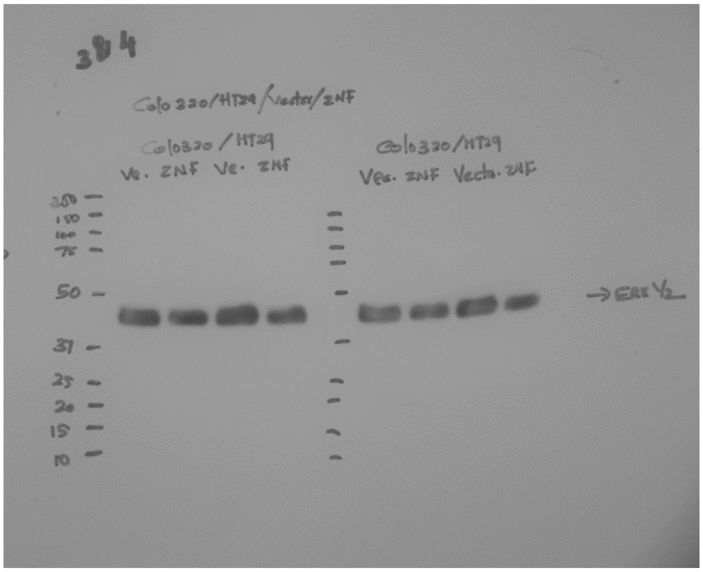
3&4
Colo320/HT29/Vector/ZNF
Colo320 / HT29
Ve. ZNF Ve. ZNF
Colo320/HT29
Ve. ZNF Vecto. ZNF
50
37
25
20
15
10
→ ERK 1/2

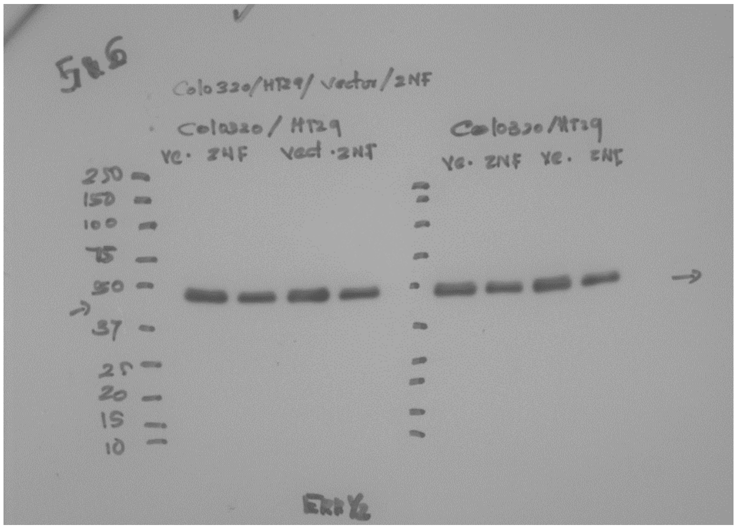
5&6
Colo320/HT29/ Vector/ZNF
Colo320 / HT29
Ve. ZNF Vect. ZNF
Colo320/HT29
Ve. ZNF Ve. ZNF
250
150
100
75
50
37
25
20
15
10
ERK 1/2

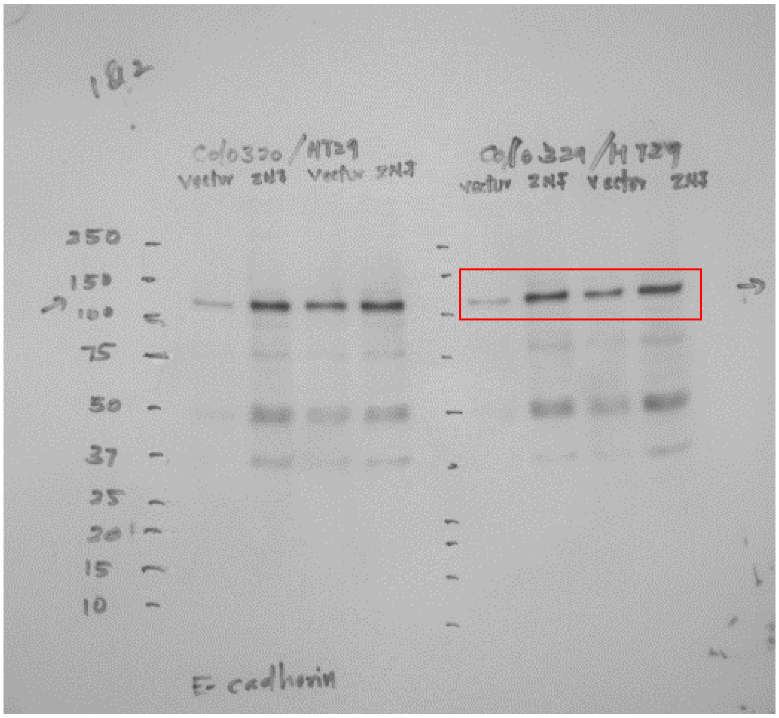
Colo320/HT29
vector ZNF vector ZNF
Colo320/HT29
vector ZNF vector ZNF
250
150
100
75
50
37
25
20
15
10
E-cadherin

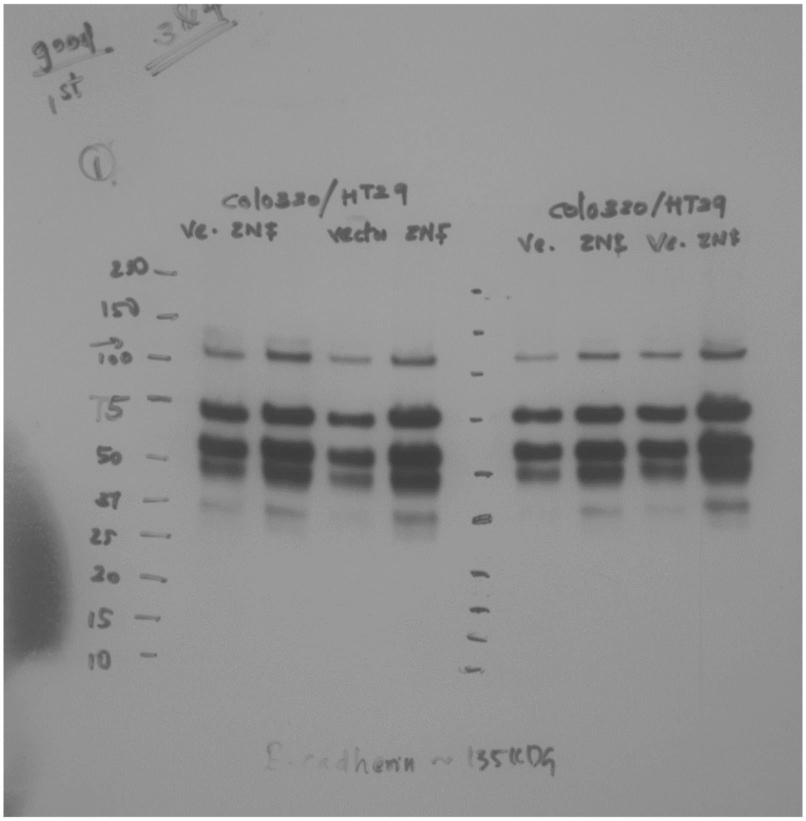
good.
1st
Colo320/HT29
Ve. ZNF vector ZNF
Colo320/HT29
Ve. ZNF Ve. ZNF
250
150
100
75
50
37
25
20
15
10
E-cadherin ~ 135KDa

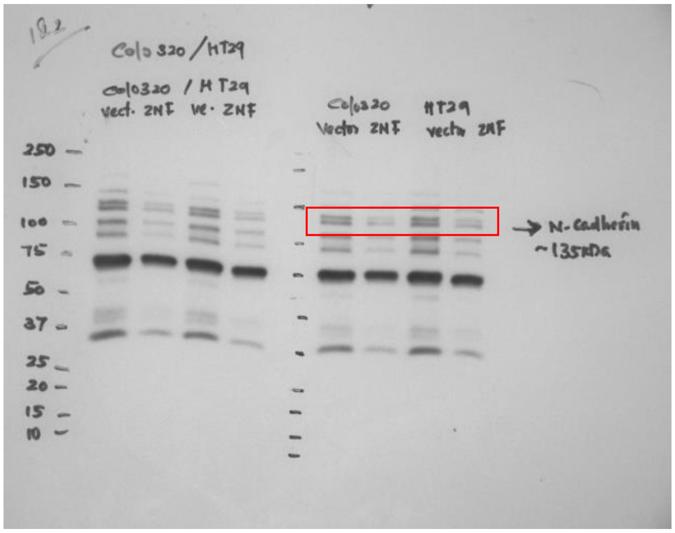

Colo 320 / HT29
Colo320 / HT29
vect. ZNF ve. ZNF
Colo320 HT29
Vector ZNF vector ZNF
250
150
100
75
50
37
25
20
15
10
→ N-Cadherin
~135kDa

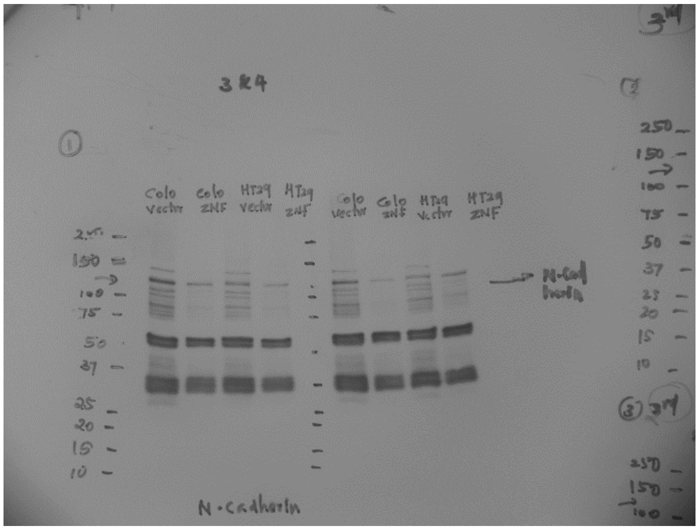

3 & 4
Colo vector
Colo ZNF
HT29 vector
HT29 ZNF
Colo vector
Colo ZNF
HT29 vector
HT29 ZNF
150
100
75
50
37
25
20
15
10
250
N-Cadherin

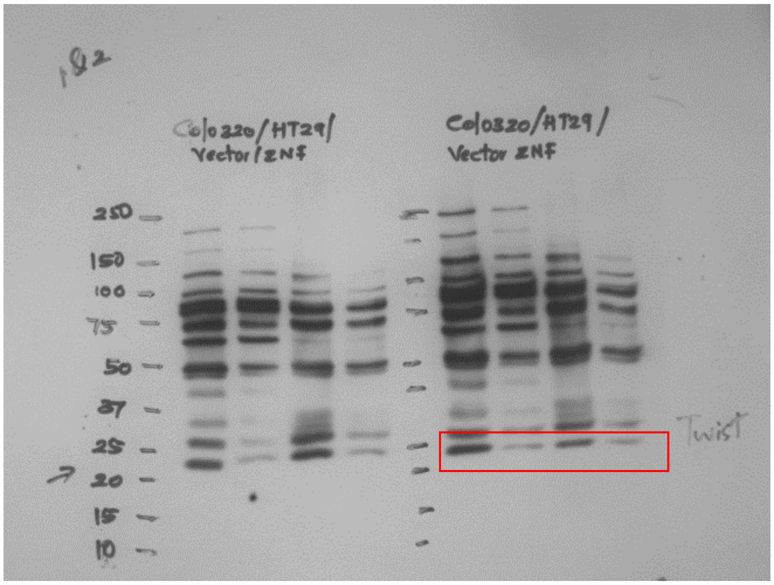
1&2
Colo320/HT29/
Vector/ZNF
Colo320/HT29/
Vector ZNF
250
150
100
75
50
37
25
20
15
10
Twist

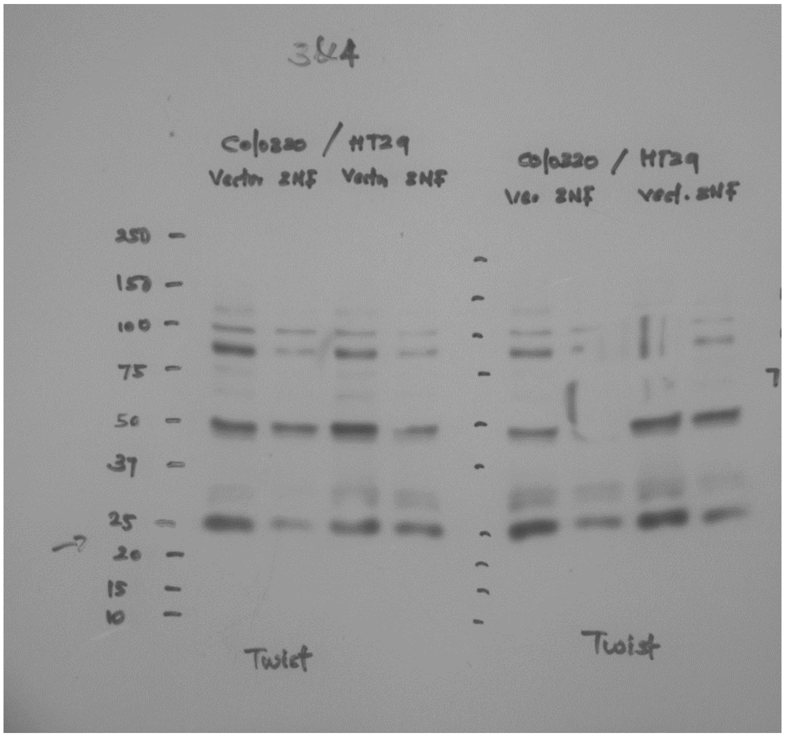
3&4
Colo320 / HT29
Vector ZNF Vector ZNF
Colo320 / HT29
250
150
100
75
50
37
25
20
15
10
Twist
Twist

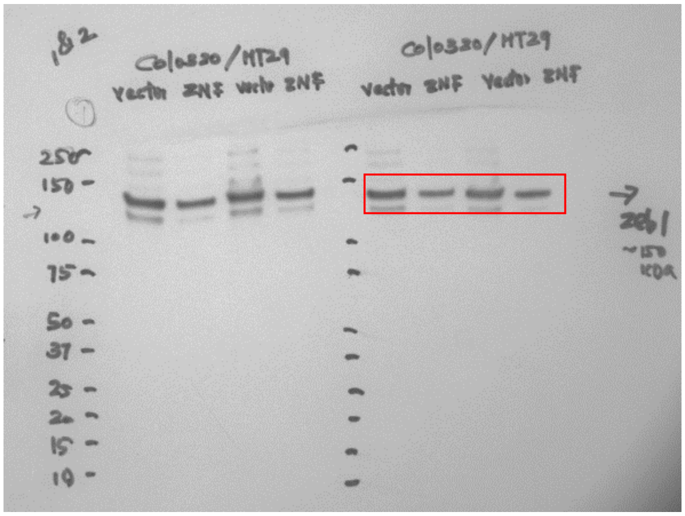
Colo320/HT29
Vector ZNF Vector ZNF
Colo320/HT29
Vector ZNF Vector ZNF
250
150
100
75
50
37
25
20
15
10
zeb1
~150 KDa

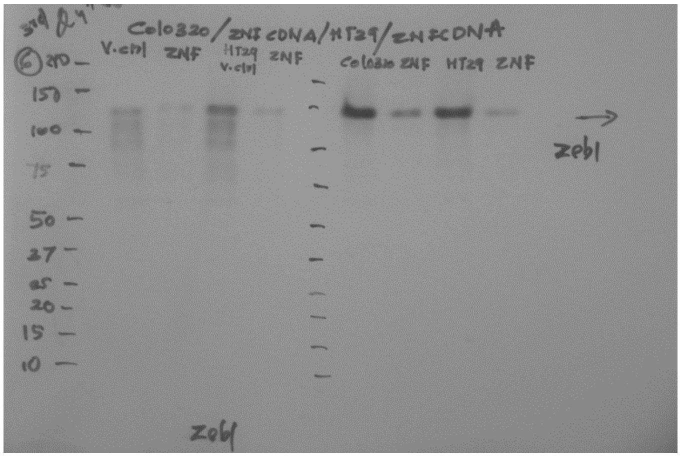
Colo320/ZNF cDNA/HT29/ZNF cDNA
V.ctrl ZNF
HT29 V.ctrl ZNF
Colo320 ZNF HT29 ZNF
250
150
100
75
50
20
15
10
zeb1
zeb1

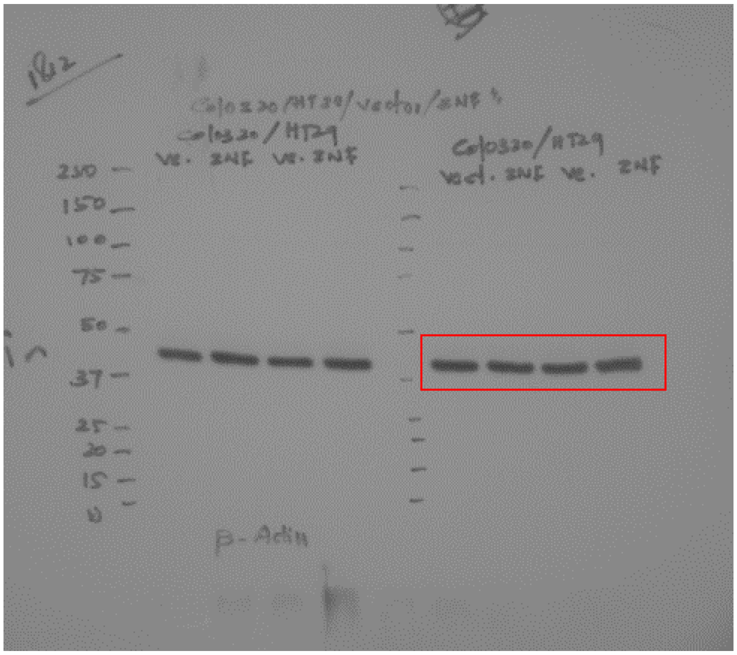

1&2
Colo320/HT29
Ve. ZNF Ve. ZNF
Colo320/HT29
Vect. ZNF Ve. ZNF
250
150
100
75
50
37
25
20
15
β-Actin

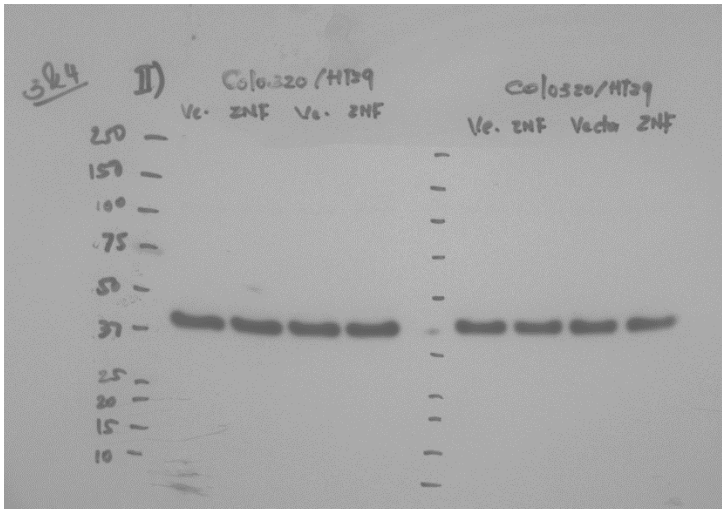

3&4
II)
Colo320/HT29
Ve. ZNF Ve. ZNF
Colo320/HT29
Ve. ZNF Vector ZNF
250
150
100
75
50
37
25
20
15
10

Figure 5A

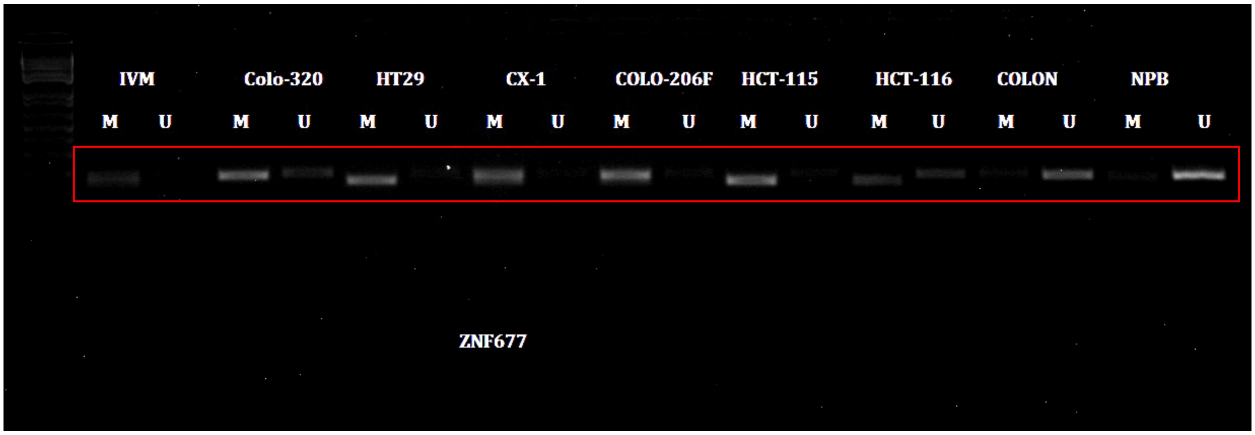

Figure 5B

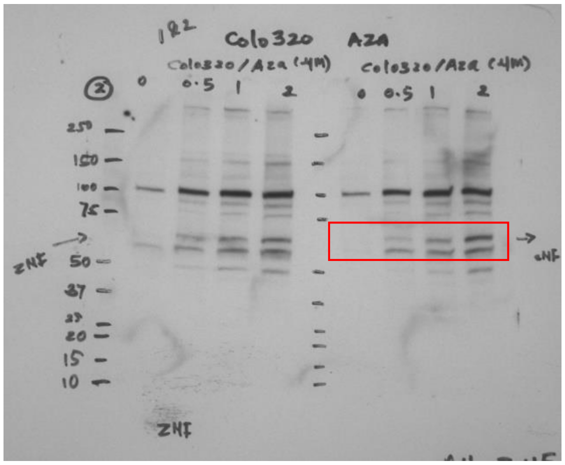

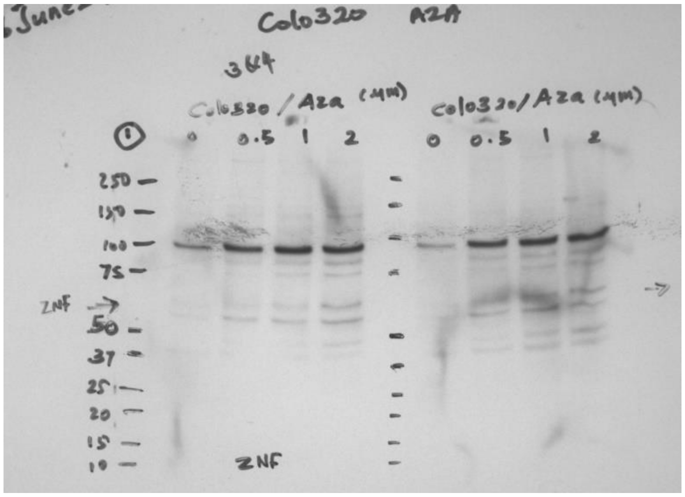
Colo320 AZA
3&4
Colo320 / Aza (uM)
Colo320/Aza (uM)
①
0 0.5 1 2
0 0.5 1 2
250
150
100
75
ZNF
50
37
25
20
15
10
ZNF

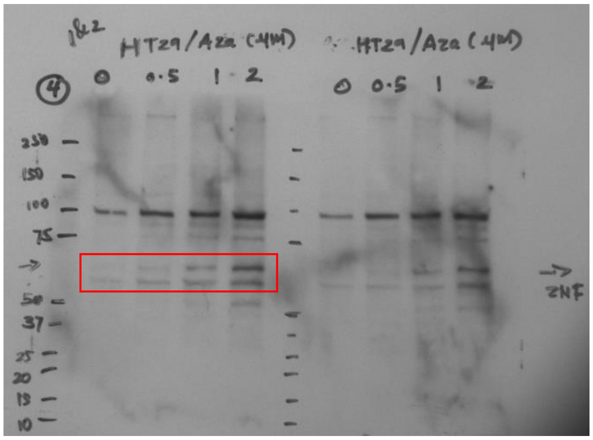
1&2
HT29/Aza (uM)
HT29/Aza (uM)
④
0 0.5 1 2
0 0.5 1 2
250
150
100
75
ZNF
50
37
25
20
15
10

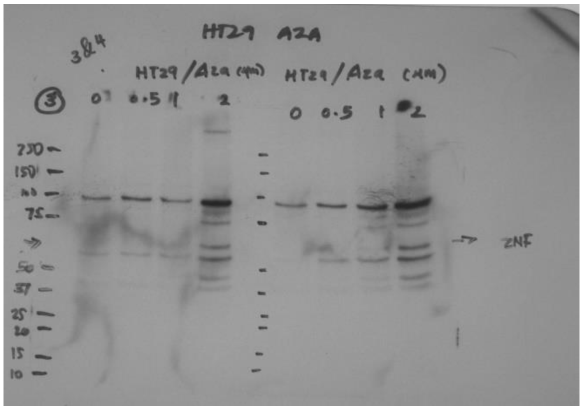
HT29 AZA
3&4
HT29/Aza (uM)
HT29/Aza (uM)
③
0 0.5 1 2
0 0.5 1 2
250
150
100
75
ZNF
50
37
25
20
15
10

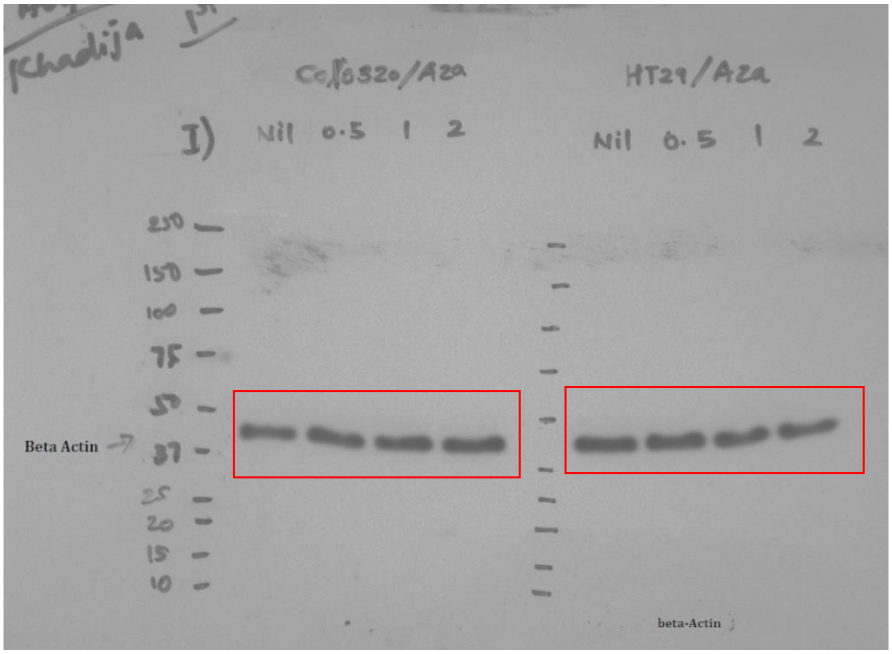
Khadija
Colo320/Aza
HT29/Aza
I)
Nil 0.5 1 2
Nil 0.5 1 2
250
150
100
75
50
37
25
20
15
10
Beta Actin
beta-Actin

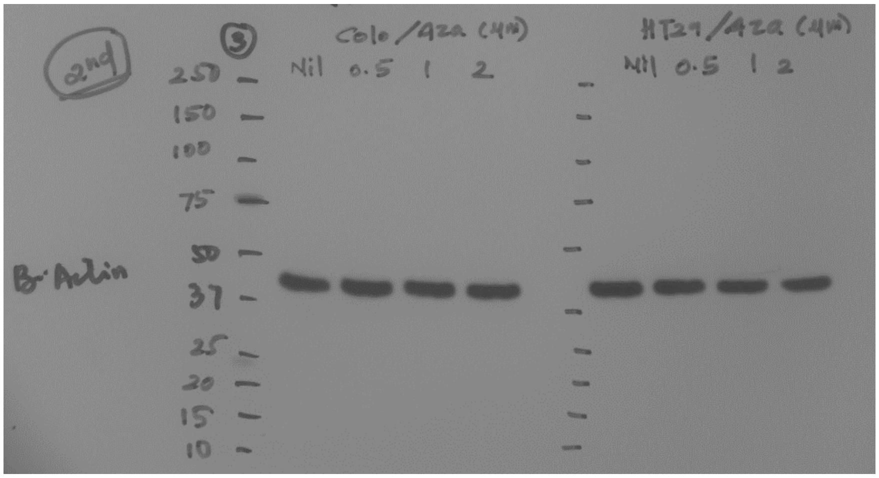
2nd
Colo/Aza (4W)
HT29/Aza (4W)
Nil 0.5 1 2
Nil 0.5 1 2
250
150
100
75
50
37
25
20
15
10
B-Actin
